# A predictive structural model for bulk metallic glasses

**DOI:** 10.1038/ncomms9123

**Published:** 2015-09-15

**Authors:** K. J. Laws, D. B. Miracle, M. Ferry

**Affiliations:** 1 School of Materials Science and Engineering, UNSW Australia, Sydney, New South Wales 2052, Australia; 2 Air Force Research Laboratory, Materials and Manufacturing Directorate, 2230 Tenth Street, Wright-Patterson AFB, Ohio 45433 USA

## Abstract

Great progress has been made in understanding the atomic structure of metallic glasses, but there is still no clear connection between atomic structure and glass-forming ability. Here we give new insights into perhaps the most important question in the field of amorphous metals: how can glass-forming ability be predicted from atomic structure? We give a new approach to modelling metallic glass atomic structures by solving three long-standing problems: we discover a new family of structural defects that discourage glass formation; we impose efficient local packing around all atoms simultaneously; and we enforce structural self-consistency. Fewer than a dozen binary structures satisfy these constraints, but extra degrees of freedom in structures with three or more different atom sizes significantly expand the number of relatively stable, ‘bulk' metallic glasses. The present work gives a new approach towards achieving the long-sought goal of a predictive capability for bulk metallic glasses.

## Abstract (web summary)

The atomic structure of metallic glasses is related to their properties such as the ability of materials to form glasses. Here, the authors develop a new approach to model the atomic structure of metallic glasses, to enable a better connection between atomic structure and glass properties.

## Graphical abstract

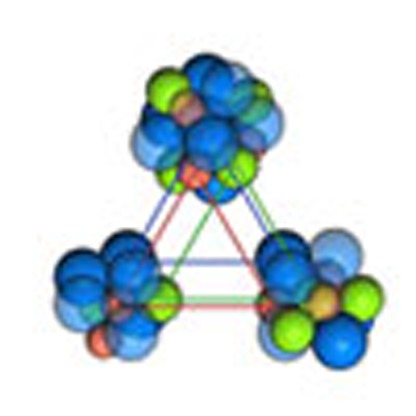

From the moment metallic glasses were discovered in 1960 (ref. 1), questions arose about their atomic structure. The dense random packing (DRP) model was introduced independently to describe the structure of monatomic liquids^2^,^3^,^4^. The metallic glass community adopted the DRP model, even though it consisted of single-sized atoms and metallic glasses always had atoms of different sizes. Attempts to put smaller atoms in the natural gaps of the DRP model^5^,^6^ were abandoned since the gaps were too small and too few to agree with metallic glasses^7^. In a dramatic break from the DRP model, the stereo-chemically defined (SCD) model used efficiently packed, solute-centred clusters with total coordination of 9 as structural building blocks for metal-metalloid glasses^8^. This model included atoms of unequal size and gave a physical basis for chemical short-range order (SRO) known to exist in metallic glasses. However, it could not explain the medium-range order (MRO) found soon after the SCD model was introduced^9^, there was never a satisfying description of how efficiently packed clusters were arranged to avoid packing frustration^10^, and it could not explain the full range of atom sizes and concentrations that produced metallic glasses. Studies clearly showed that metallic glass structures were, indeed, efficiently packed^11^,^12^,^13^,^14^, but none were able to explain how glass structures accomplished this feat. Reviews of the first 30 years of metallic glass structural modelling are available^15^,^16^,^17^.

Thus, 40 years after their discovery^1^, three seminal questions for metallic glasses remained: what is their atomic structure; how does structure influence glass-forming ability (GFA); and what chemical interactions are needed for good GFA? Great strides have been made in the past 10 years in resolving the first question. By extending ideas from the SCD model, a new model that uses efficiently packed solute-centred atom clusters as structural building blocks has been established^18^,^19^, and quantitative predictions of SRO^20^, MRO^21^ and density^22^ have validated and refined this efficient cluster-packing (ECP) model. This model gives new ways to interrogate atomic simulations^19^, opens new ways to analyse experimental data^23^ and has inspired new experimental techniques^24^. Bulk metallic glasses (BMGs, glasses with the smallest amorphous dimension ≥1 mm) were first discovered nearly 25 years ago^25^,^26^, but finding new BMGs still requires tedious trial-and-error methods. However, the ECP model has inspired the discovery of new BMGs without full recourse to empiricism^27^,^28^,^29^,^30^. Efficient local atomic packing around the minority (solute) atoms and a preference for bonds between unlike atoms gives the physical motivation for structure-forming clusters^16^. Efficient packing is achieved for specific radius ratios between solute and solvent atoms, 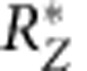, that give solute-lean clusters with a central solute atom surrounded by *Z* solvent atoms^31^. The most stable glasses are typically solute rich, where the solute atom first shell contains both solute and solvent atoms. The ECP model gives a good account of both solute-lean and solute-rich structures^32^. Additional details of this model are given in ref. 21.

In spite of this progress, current structural models are not predictive. The ECP model describes the structure once a glass is formed, but it cannot predict which structures will have good GFA (defined here as the maximum fully amorphous thickness or diameter produced by quenching from the liquid), which will form marginal glasses, and which will not produce glasses at all. Important questions that may give insights into GFA remain unanswered. Of the dozen 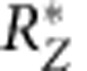 values that give efficient local atomic packing (9≤*Z*≤20), there is no explanation why five values are strongly preferred, giving all of the most stable binary glasses^32^. Even more troubling, the basis for preferred radius ratios that underpins the ECP model seems to disappear in solute-rich glasses, where efficient filling of the first coordination shell can be achieved at any radius ratio by adjusting the constitution of the first shell. The structural features that are essential for metallic glasses to compete with crystalline phases thus remain a mystery.

Here we solve three persistent problems to give new insights into this seminal question: we identify a new family of structural defects that discourage metallic glass formation; we learn how to enforce efficient packing around every atom in the glass simultaneously; and we determine the relative atom sizes and concentrations and the distinct clusters that they enable for self-consistent compositions. These ideas are consistent with earlier work on the ECP model, and solution of these problems emphasizes atomic structures that are ‘solute-rich' (have sufficiently high concentrations so that cluster first shells must contain all of the atom species present) or where the smaller atoms are the majority species. Such structures represent a large number of BMGs, and their inclusion was not always clear in earlier developments of the ECP model. The present work thus gives a more predictive, robust and complete description of metallic glass atomic structures.

## Results

### Glass suppression via super-substitutional atomic defects

**Figure 1:**
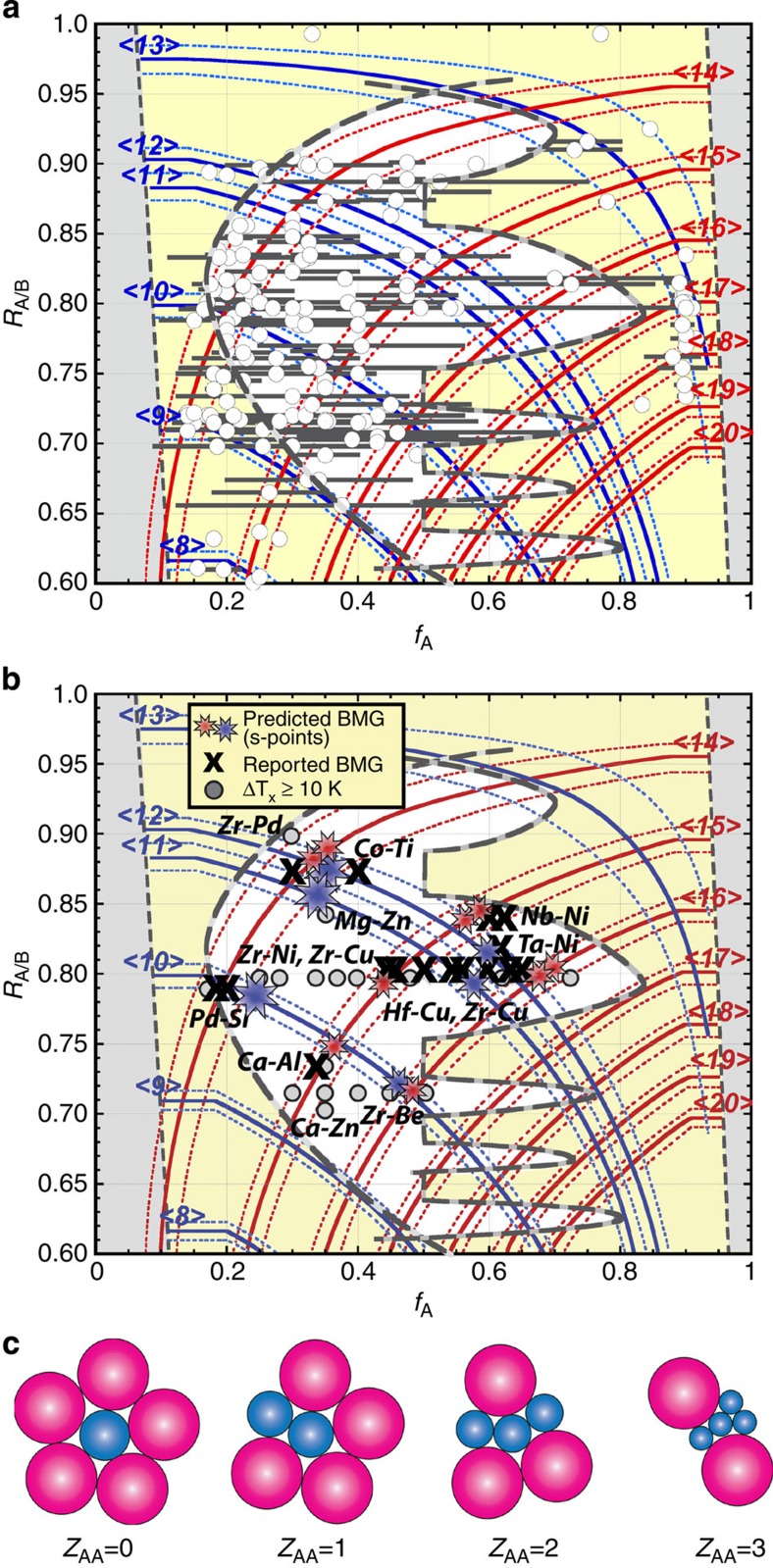
Efficient cluster-packing curves. Efficiently packed (P=100±1%) A- and B-centred clusters of fixed total coordination numbers <*Z*_A,tot_> (blue lines) and <*Z*_B,tot_> (red lines) as a function of *R*_A/B_ (nominal radius ratio) and *f*_A_ (A atom fraction). These curves are superimposed with (**a**) all binary metallic glasses (empty circles). Horizontal bars show the composition range for each system. Regions excluded by a minimum solute criterion are shown by grey regions and relative atom sizes and concentrations discouraged by competing crystalline defects are shown by yellow areas. (**b**) Binary BMGs (black crosses) and glasses with Δ*T*_*x*_>10 K (grey circles) occur near S-points (eight-point stars), where packing is efficient around both clusters simultaneously. (**c**) *R*_A/B_ decreases with increasing *Z*_AA_ and *f*_A_ in clusters of fixed <*Z*_A,tot_>, since the size difference between A and B atoms has to increase to maintain efficient packing as smaller A atoms replace larger B atoms in the 1st shell. In B-centred clusters, the size difference between A and B atoms has to decrease (*R*_A/B_ increases) with increasing *f*_A_. Data are taken from refs 32, 51. The atom radii used in the present work are given in Supplementary Table 1.

To predict which alloys will form glasses, we must first understand why some alloys do not. We consider binary systems of A and B atoms with radii *r*_A_<*r*_B_ and A atom fraction *f*_A_. Atom size-concentration space is bounded by 0≤*f*_A_≤1 and radius ratios 0≤*R*_A/B_≤1, where *R*_A/B_=*r*_A_/*r*_B_. Metallic glasses are generally limited to 0.6≤*R*_A/B_≤1 due to the radii of metallic elements^32^. Glasses with *f*_A_>0.6 account for 40% of atom size-composition space but fewer than 20% of binary metallic glasses, so that some unknown feature seems to discourage binary glasses when the small atom is the majority species (Fig. 1a).

Binary glasses are forbidden below a minimum solute fraction, *f*_A, min_=1/(*Z*+1), where each solute is surrounded only by solvent atoms and each solvent touches only one solute. Glasses can form above *f*_A, min_, but they are discouraged between *f*_A, min_ and the higher solute fraction, *f*_crit_, that is needed for glasses to compete more effectively with crystals. Solute atoms produce local elastic strains in crystal structures that compete with the glass, and a glass can form when the volume strain throughout the competing crystal reaches a critical value, *ɛ*_crit_ (refs 33, 34). Solutes with large local strains can be far apart (have low *f*_crit_), while solutes with smaller strains must have higher concentrations to reach *ɛ*_crit_. The magnitude of elastic strains depends on the type of defects. Substitutional defects dominate when *R*_A/B_>0.81 and A atoms are solutes (left-hand side of Fig. 1a)^33^,^34^ and interstitial defects occur when *R*_A/B_<0.81 (ref. 34). The critical concentration to destabilize competing crystals, *f*_crit_, decreases as *R*_A/B_ decreases, reaches a minimum near *R*_A/B_=0.81, then increases with further reduction in *R*_A/B_ (Fig. 1a). For B-atom solutes (right-hand side of Fig. 1a), earlier approaches only considered substitutional defects where a solute replaced a single solvent atom^33^,^34^. This gives *f*_crit_ values that decrease continuously as *R*_A/B_ decreases (*f*_crit_=1–*f*_A_ when B is the solute), and a large portion of the sparsely populated size-composition space for *f*_A_>0.6 remained unexplained.

Here we show that large solutes may replace more than one solvent atom. These super-substitutional defects (see Methods) have lower strains—and higher *f*_crit_ values—than single substitutional defects near the radius ratios where these defects form. Five super-substitutional *f*_crit_ peaks now discourage most size-composition combinations on the right-hand side of Fig. 1a. This new family of atomic defects explains for the first time why the large portion of atom size-composition space at *f*_A_>0.6 produces relatively few metallic glasses.

### Simultaneous efficient packing in binary metallic glasses

We now turn our attention from excluded and discouraged glasses to predicting those that are most likely to form metallic glasses. Packing efficiency has long been considered important^12^,^14^,^35^ and local packing efficiency around solute atoms is the basis for the ECP model. The concept that packing may be efficient around both solute and solvent atoms^36^ was not included in the ECP model earlier due to several problems. The packing efficiency bounds (±10%) were too broad to be meaningful, validation was given for only a small number of marginal glasses, and the earlier work did not give a physical model proving that efficient packing could be achieved around all atoms simultaneously. Broad statements have suggested that local packing is efficient around solute and solvent atoms^37^, but this remains an unproven concept that still requires a compelling physical model.

Here we develop a new approach to enforce efficient local packing around all atom species that overcome these concerns. We start by plotting efficiently packed cluster curves for each total coordination number (Fig. 1). These curves hold constant the packing efficiency (*P*^i^) and the total number of atoms in the first shell of *i*-centred clusters (*Z*_i,tot_). Composition is changed by adjusting the partial coordination numbers, *Z*_ij_, given as the number of *j* atoms in the first shell of *i*-centred clusters. The atom fractions of structures derived from these clusters are


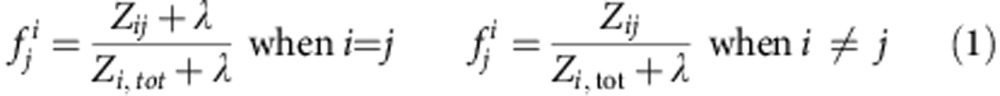


where *j* is the atom for which the composition is given. *λ* is the number of solute atoms on solute sites per cluster (1≤*λ*≤4, and the value of *λ* has only a minor effect in the present work). We adjust *R*_A/B_ for each *Z*_ij_ to keep clusters efficiently packed (Fig. 1 inset). The radius ratios in these efficiently packed A- and B-centred clusters are (see Methods)


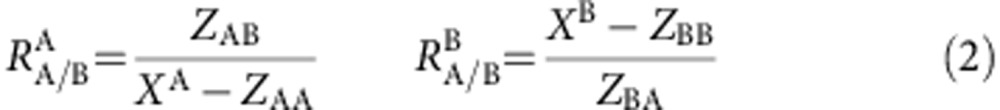


where 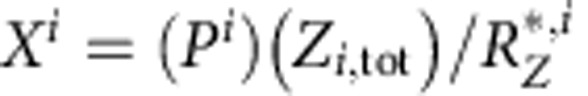 and 
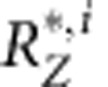
 is the radius ratio that gives ideal packing for an integer number *Z* of identical atoms in the first shell of *i*-centred clusters^31^.

All binary BMGs essentially fall within *P*=100±1% of at least one cluster (Fig. 1b), as do glasses discouraged by substitutional, interstitial and super-substitutional defects (Fig. 1a). Where two curves intersect, *f*_A_, *R*_A/B_ and *P* are the same in A-centred and B-centred clusters simultaneously. This is a remarkable convergence, and some of the most stable binary BMGs (Pd_100−*x*_Si_*x*_, 18≤*x*≤20, Cu_64_Zr_36_ and Cu_65_Hf_35_) are nearly coincident with two such intersections. However, there is no compelling correlation between these intersections and the full set of binary BMGs (Fig. 1b). Below we show that such a convergence can be achieved in some (but not all) binary systems, but at different atom sizes and compositions than those indicated by curve intersections.

### Structural self-consistency in binary metallic glasses

Two conditions must be satisfied rigorously in physical systems. First, the nominal radius ratios of *i*-centred clusters, 
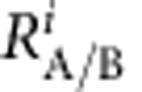
, must be identical in both A- and B-centred clusters


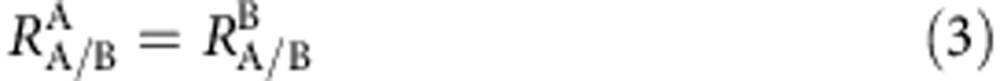


Structural self-consistency is the second requirement. Self-consistency recognizes that the number of A–B bonds in a structure is fixed, so that the number of A–B bonds ‘originating' from A atoms equals the number of A–B bonds ‘originating' from B atoms. This gives the self-consistency equation


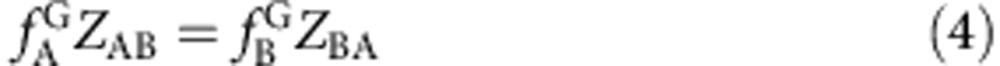


where 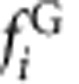 is the global atom fraction of element *i*. equation (4) is used in diffraction analyses^38^, but has not been used previously in structural modelling. To determine the structures that allow efficient packing around both atoms in the same structure, we solve equations (3 and 4) simultaneously (see Methods). The results are given in Table 1 and Fig. 1b. More detailed discussion of excluded, discouraged and preferred atom sizes and concentrations is given in Supplementary Notes 1–3 and Supplementary Figs 1–3.

### S-points and the most stable binary metallic glasses

Discrete atom sizes and concentrations that simultaneously satisfy equations (3 and 4) are called S-points. These are the only points in binary size-composition space where packing is efficient around both atoms simultaneously. Each <*Z*_A,tot_, *Z*_B,tot_>curve intersection has two solutions. A-reference S-points (blue stars in Fig. 1b) set the A atom fraction in the glass, 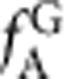, to the composition of A-centred clusters, 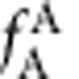. B-reference S-points (red stars in Fig. 1b) give solutions where 
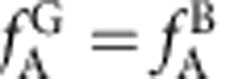
. Some S-points are near the corresponding <*Z*_A,tot_, *Z*_B,tot_> intersection, but some are not. Some S-points are close together and some S-points are suppressed by the *ɛ*_crit_ criterion, so that only about a dozen distinct S-points exist. A graphical description of how S-points are determined is given in Supplementary Note 3 and Supplementary Fig. 3.

Pd–Si glasses are by far the most stable binary BMGs, suggesting that some S-points have better GFA than others. The local compositions of A- and B-centred clusters, 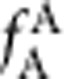 and 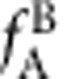, are not the same in binary glasses (see Table 1). This is because self-consistency requires partial coordination numbers that give different compositions in A- and B-centred clusters. However, how can A- and B-centred clusters have different local compositions in the same glass? The cluster compositions in equation (1) are only preferences. For example, equation (1) implies that all *i*-centred clusters are identical. While particular clusters dominate a given glass, a range of quasi-equivalent clusters can occur^19^. Further, the number of occupied β and γ sites (structural sites between an octahedron and a tetrahedron of clusters, respectively^21^) in a structure may vary. Finally, the ECP model assumes that β and γ sites are vacant or are occupied by solute atoms^21^, but solvent atoms may also occupy these sites. The curves in Fig. 1 thus show preferences and do not give exact glass compositions.

The composition difference between A- and B-centred clusters gives a chemical frustration that needs to be minimized. We propose that S-points with the best GFA will have the smallest composition difference,


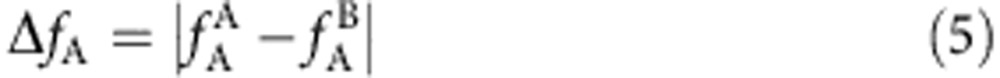


Δ*f*_A_ is the degree to which a structure must adjust from preferences in equation (1) to achieve efficient packing around both species and to satisfy self-consistency. Compositions cannot adjust by changing *Z*_AB_ or *Z*_BA_, which are fixed by equations (3 and 4), but can change by altering the number of *λ* sites occupied or by changing the types of atoms on *λ* sites. A change of one atom per cluster gives a composition change of ±1/(*Z*_*i*,tot_+1) as a benchmark against which Δ*f*_A_ can be compared. The three best S-points have Δ*f*_A_ below this benchmark and are shown by larger stars in Fig. 1b. Table 1 sorts S-points by increasing Δ*f*_A_. Pd–Si glasses have the best GFA of any binary BMG and have the second smallest Δ*f*_A_. Few glasses have been made near the remaining two preferred S-points (compare Fig. 1a,b), suggesting a good place for future exploration.

There has never been an explanation for why the most common *R*_A/B_ in binary glasses is near 0.80 and why BMGs form over a nearly continuous composition range of 0.45≤*f*_A_≤0.65 at this radius ratio^32^. Four S-points (<10,15>_B_, <11,15>_A_, <11,16>_B_ and 12,16>_B_, where the subscript indicates the reference structure) fall within an exceptionally narrow range of *R*_A/B_=0.798±0.007 and span compositions from 0.44≤*f*_A_≤0.70. Over half the known binary BMGs fall between these S-points. This shows that glasses can form between S-points that have the same *R*_A/B_ values and are compositionally close to each other. The GFA of such glasses may depend on the compositional distance from the bounding S-points. Thus, relatively stable glasses form between <10,14>_A_ and <10,15>_B_ S-points in the range 0.25≤*f*_A_≤0.40 (Fig. 1), but they do not form BMGs because the composition distance between the bounding S-points is large. The radius ratio of Cu–Zr and Cu–Hf glasses (*R*_A/B_=0.797) is very close to the radius ratio of these S-points, and the widest composition range allowed by the *ɛ*_crit_ criteria also occurs near *R*_A/B_=0.80. Together, these features explain for the first time why alloys with *R*_A/B_≅0.80 are by far the most common and the most stable binary glasses.

It seems fortuitous that the favoured radius ratio *R*_A/B_≅0.80 is so close to the ideal radius ratio 
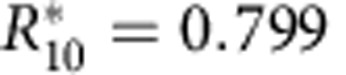
 for efficient packing of <10> clusters with only B atoms in the first shell. The present work shows that solute-rich clusters with *Z*_*i*,tot_=11, 12, 14, 15 and 16 are also produced at this radius ratio (Fig. 1), consistent with experimental *Z*_*ij*_ data^20^.

Agreement between predicted and observed binary glasses is good. No glasses occur in excluded regions. Glasses discouraged by atomic defects in competing crystals (yellow regions of Fig. 1) are relatively few, generally have one efficiently packed cluster (Fig. 1a) and are always marginal glasses (amorphous thickness, *t*_a_<1 mm with a difference between crystallization and glass transition temperatures, Δ*T*_x_≅0 K). S-points are uncommon, consistent with the restricted number of BMGs and near-BMGs (*t*_a_<1 mm but Δ*T*_x_≥10 K). Each S-point has BMGs or near-BMGs (or both) nearby and all BMGs and near-BMGs are close to S-points. A most favoured S-point (<10,14>_A_) gives the only exceptional binary BMG (Pd–Si glasses with *t*_a_>5 mm). The present model thus not only predicts whether or not a glass will form, but indicates whether the glass will be marginal, near-BMG, BMG or an exceptional BMG. The results in Table 1 for *P*^A^=*P*^B^=100% are not substantially changed by varying *P*^A^ and *P*^B^ within ±1%, but agreement degrades outside these bounds. Thus, essentially all of the most stable binary metallic glasses discovered in over 50 years of extensive experimentation are well-represented by the present predictions.

### Simultaneous efficient packing and ternary BMGs

**Figure 2:**
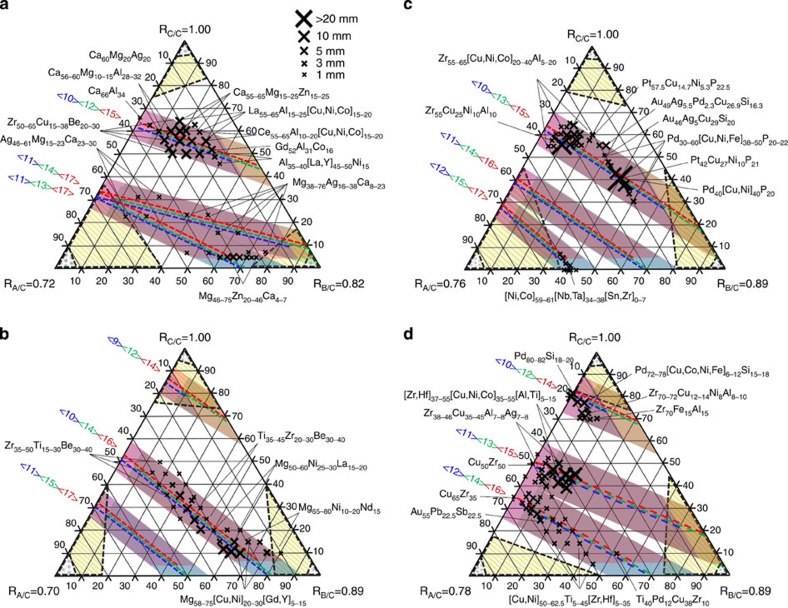
Ternary diagrams indicating simultaneous efficient packing (*P*=100±2%) of A- (blue-dashed lines and shading), B- (green-dashed lines and shading) and C- (red-dashed lines and shading) centred clusters at fixed <*Z*_A,tot_>, <*Z*_B,tot_>, <*Z*_C,tot_> for different radius ratios. (**a**) *R*_A/C_=0.72, *R*_B/C_=0.82; (**b**) *R*_A/C_=0.70, *R*_B/C_=0.89; (**c**) *R*_A/C_=0.76, *R*_B/C_=0.89 and (**d**) *R*_A/C_=0.78, *R*_B/C_=0.89. Exclusion zones based on binary limits are indicated in yellow. Reported BMG compositions (black crosses) are also shown. Data are taken from ref. 52. The atom radii used in the present work are given in Supplementary Table 1.

The present approach shows a good ability to predict binary GFA, but the number of binary BMGs is small. There are many ternary BMGs, and so we extend these concepts to ternary glasses to better explore their robustness. We use systems of A, B and C atoms with radii *r*_A_<*r*_B_<*r*_C_ and radius ratios *R*_A/C_=*r*_A_/*r*_C_, *R*_B/C_=*r*_B_/*r*_C_ and *R*_C/C_=1. Some glasses with four or more chemically distinct species have only three significantly different atom sizes. For example, Zr–Al–[Cu,Ni] and Pd–[Ni,Cu]–P are generally considered as quaternary glasses, but since *r*_Ni_≅*r*_Cu_ these glasses have only three significantly different atom sizes. Such glasses are considered as structural ternary glasses, since equal-sized atoms will occupy similar structural sites. For convenience, we show atoms of nearly equal size in brackets. In the same way, glasses with only four significantly different atom sizes are considered structural quaternary glasses, regardless of the number of chemical species. The relative atom sizes and concentrations of many ternary BMGs are plotted in Fig. 2.

Predicted relative atom sizes and concentrations for ternary BMGs are found using the same approaches for binary glasses. Excluded and discouraged structures fill ternary corners using *f*_crit_ values from Fig. 1. For simplicity, a straight line connects pairs of *f*_crit_ values on the ternary edges. Simultaneous efficient packing in ternary systems occurs along straight lines (see Methods). The bands in Fig. 2 show packing efficiencies of 100±2% for each <*Z*_A,tot_, *Z*_B,tot_, *Z*_C,tot_> structure. Self-consistency significantly limits the number of binary atom sizes and concentrations with efficient packing around all species, but self-consistency is always achieved when the cluster composition is the same as the glass composition in ternary glasses due to extra degrees of freedom (see Methods). Thus, discrete S-points are not needed in more complex glasses and all of the ternary glass structures shown in Fig. 2 satisfy self-consistency. Details of the ternary systems in Fig. 2 are given in Supplementary Note 4 and Supplementary Table 3.

The present model is validated by comparing predicted atom sizes and concentrations with more than 230 reported ternary BMGs in 57 chemically distinct systems. Essentially all of these BMGs have atom sizes and concentrations where packing is simultaneously efficient around all atoms (all three bands overlap in Fig. 2). Over 40 of these ternary BMGs and 20 quaternary BMGs^27^,^28^,^29^,^30^ were first predicted using some of the present ideas, indicating their predictive capabilities. To further establish their predictive abilities, we used the present ideas to find 35 new Mg-based BMGs (Supplementary Table 4). These new discoveries include ternary systems never reported before: Mg–Ag–Yb; Mg–Pd–Ca and Mg–Pd–Yb. We also find the highest glass transition temperature for any Mg-based BMG (485 K for Mg_67.5_Pd_25_Ca_7.5_). To put this achievement in perspective, thousands of compositions are usually needed using conventional trial-and-error methods to discover one new BMG. Combinatorial methods accelerate this process, but the failure-to-success ratio is still over 1,000 to 1 (ref. 39). The present model dramatically improves these odds—we produced 35 new BMGs from 44 predicted alloys.

**Figure 3:**
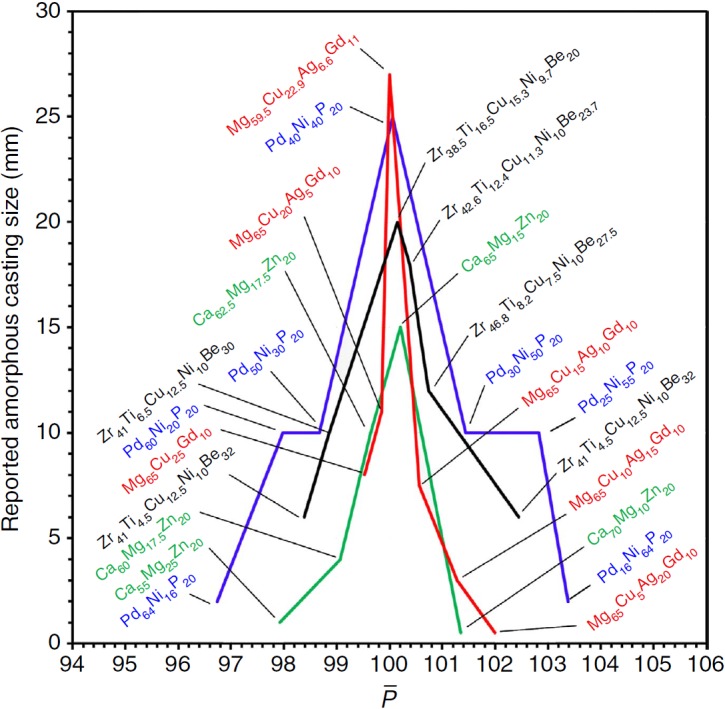
Reported maximum amorphous thickness versus the mean packing efficiency around all atoms in the structure, 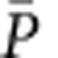. Data are shown for several ternary and quaternary glasses. The maximum thickness is found when 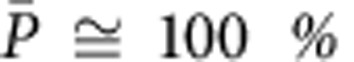 for each of the glasses. The data are taken from refs 53, 54, 55, 56, 57, 58, 59, 60.

These ideas suggest that the best BMGs will have atom sizes and concentrations that fall nearest the ideal packing lines in Fig. 2. The largest known glass diameter is 80 mm for fluxed Pd_42.5_[Cu_30_Ni_7.5_]P_20_ (ref. 40), an alloy that lies perfectly on ideal packing lines for <10,13,15> structures. Since identical structures can occur in different chemical systems, Fig. 2 mixes contributions to glass stability from structure (efficient packing) and chemical effects. To isolate the structural contribution, we consider ternary BMGs with different concentrations of the same elements. The glasses must fall in a relatively narrow composition band to avoid transitioning to a different structure. We plot glass thickness against the mean packing efficiency around the *n* constituent atoms, 
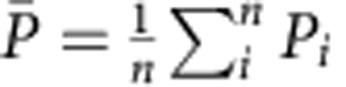
 (Fig. 3). Pd–Ni–P and Ca–Mg–Zn ternary glasses both show the maximum amorphous thickness when the mean packing efficiency is closest to 100%. The amorphous thickness drops significantly as atoms become over-packed (
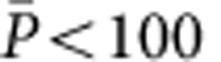
%) or under-packed (
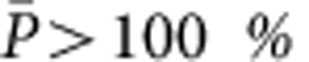
). This figure shows a sharp ‘cusp' behaviour similar to the effect of composition on GFA documented for many other systems^41^.

Preferred atom sizes and concentrations in Fig. 2 with no reported BMGs may be regions for future exploration. However, some efficiently packed structures may lack combinations of ‘real-world' atoms to give the radius ratios and chemical interactions needed to form BMGs. Thus, BMGs might not be produced in every preferred region.

### Simultaneous efficient packing and higher order metallic glasses

We extend analysis to structural quaternary and quinary BMGs—glasses with four or five different atom sizes, respectively, and with as many as seven different elements. Alloys from common quaternary and quinary systems (Supplementary Tables 5 and 6) were used to validate the present ideas. The packing efficiencies of considered glasses all have 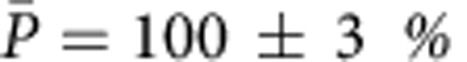. It's difficult to visualize and plot results for quaternary and quinary glasses as was done in Figs 1 and 2 for binary and ternary glasses (Supplementary Note 5 and Supplementary Fig. 4). We plot glass thickness versus 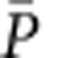 for quaternary glasses made of the same elements. Comparisons are shown in Fig. 3 for Zr–Ti–[Cu,Ni]–Be and Mg–Cu–Ag–Gd. The results are generally the same as for ternaries—BMGs occur when 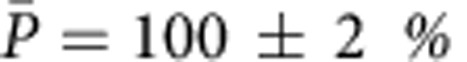 and the best GFA is achieved when 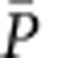 is closest to 100%.

### Challenging new ideas

Equal-sized spheres are frustrated in three dimensions, so that packing is non-ideal around every atom^42^. Thus, the distance between symmetrically-placed spheres in the first shell of an icosahedron is 5% larger than the distance to the icosahedron centre. This frustration can be overcome by packing tetrahedra of equal-sized hyperspheres on the curved surface of a four-dimensional sphere^42^,^43^, but adding unequal spheres is a much simpler three-dimensional approach to reduce frustration. For example, the gaps in an icosahedron disappear when the central sphere has a radius that is 0.9022 that of the surrounding spheres, eliminating packing frustration around the central sphere. Structures built from such efficiently packed clusters are more efficiently packed than equal-sized spheres but remain frustrated, since packing around the first shell atoms is still inefficient. This frustration is apparent by the gaps between clusters–called β and γ sites in the ECP model. In principle, packing frustration can be further reduced by filling β and γ sites, but these sites are typically smaller than metallic atoms so that filling these sites pushes the surrounding clusters apart and actually decreases global packing efficiency^22^. Thus, unequal spheres can reduce packing frustration relative to systems of equal-sized spheres, but most atoms remain inefficiently packed. This example uses iscosahedra—the same is true for structures of efficiently packed clusters with different total coordination numbers.

**Figure 4:**
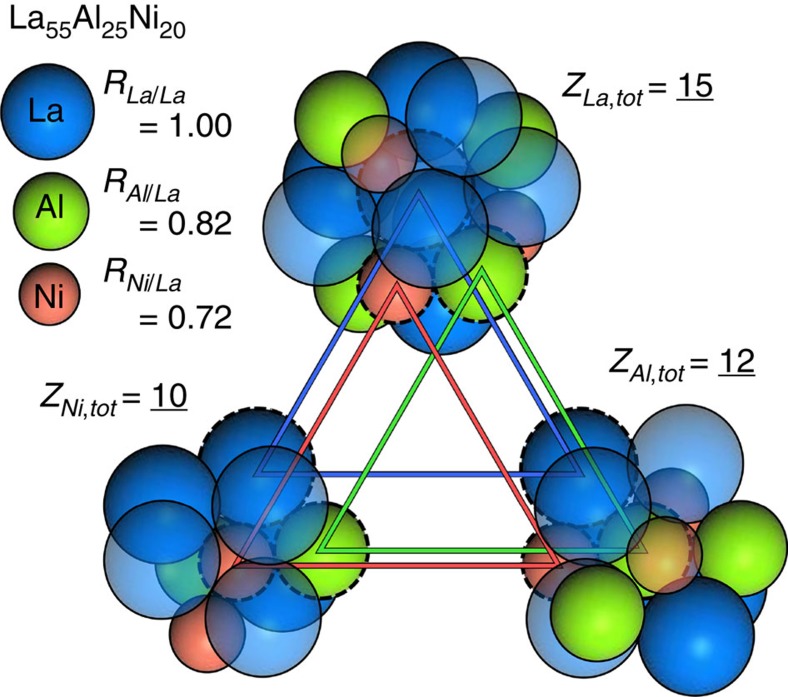
Three efficiently packed, inter-penetrating, self-consistent clusters in the Al_25_La_55_Ni_20_ BMG. The three atoms outlined by dashed lines and connected by triangles are each the centres of one cluster and are in the first shells of the other two clusters, and so are common to all three clusters. The partial and total coordination numbers are: *Z*_AlAl_=2, *Z*_AlLa_=7, *Z*_AlNi_=3, *Z*_Al,tot_=12 for the Al-centred cluster; *Z*_LaAl_=4, *Z*_LaLa_=8, *Z*_LaNi_=3, *Z*_La,tot_=15 for La-centred clusters; and *Z*_NiAl_=3, *Z*_NiLa_=6, *Z*_NiNi_=1, *Z*_Ni,tot_=10 for Ni-centred clusters. These partial coordination numbers are self-consistent, allowing clusters to inter-penetrate with local (cluster) compositions that equal the bulk composition. The relative atom sizes combine with the glass composition and partial coordination numbers to give efficient local packing around all three atom species simultaneously.

Here we further reduce packing frustration and increase global packing efficiency by establishing the specific conditions for efficient packing around all atoms simultaneously in systems of unequal spheres. Simply stated, if every atom is efficiently packed locally, then the structure may be more efficiently packed globally. This becomes apparent by realizing that atoms in the first shell of one efficiently packed cluster are the centres for other efficiently packed clusters (Fig. 4). Simultaneous efficient packing and self-consistency are achieved only on average throughout the structure, since some variability in actual partial coordination numbers, and hence in local packing efficiency, is expected due to kinetic constraints. Using atomic volumes and measured densities, global packing fractions above 0.70 and as high as 0.76 are typical for the most stable binary metallic glasses^22^. The face-centred cubic (fcc) cluster-packing scheme of the ECP model is still an essential feature, since it gives the medium-range atomic order observed in metallic glasses^18^,^21^,^44^.

Another advance is a description of the origin and impact of compositional frustration in metallic glasses. Compositional frustration cannot be avoided in binary glasses due to the limited degrees of freedom, and structures that minimize this frustration give the best binary glasses. Extra degrees of freedom in structures with three or more different atom sizes overcome this limitation, so that the composition around all atom types can simultaneously match the bulk composition. The GFA of ternary and higher order glasses is consequently improved. While compositional frustration is not required in complex glasses, it may nevertheless exist due to kinetic constraints from quenching.

The present work introduces new ways to think of metallic glass structures. The ECP model has the simple view that an atom occupies either a solute site at a cluster centre (α, β or γ) or a solvent site in the cluster first shell (Ω). Here we fundamentally change this simple description by showing that an atom satisfies requirements for both of these sites at the same time. Thus, an atom that is the centre of one cluster is also in the first shell of other atoms. While this seems self-evident, none of the earlier structural models were able to formalize this dual identity.

These ideas are illustrated in Fig. 4 for the Al_25_La_55_Ni_20_ BMG. The three constituent clusters are all inter-connected and have partial coordination numbers that combine with the relative atom sizes to give efficient local atomic packing. Not only are the partial coordination numbers self-consistent, but their arrangements are also self-consistent. This is seen by careful inspection of Fig. 4, which shows that any atom site common to two clusters is occupied by the same atom. Figure 4 also illustrates the surprising result that the preferred SRO around each atom type (reflected by partial coordination numbers and cluster composition) matches the bulk composition. This is unusual, since strong SRO is usually associated with chemical heterogeneity. For example, the local solute concentrations of metalloid-centred clusters in metal-metalloid glasses are typically half the bulk concentration. Here we show that strong SRO is achieved with essentially no local chemical heterogeneity in ternary and more complex glasses. Figure 4 illustrates the elegant inter-connections that allow structures to achieve this challenging set of conditions simultaneously.

### Structure, kinetics and chemistry

The present work seems to suggest that GFA can be predicted without considering kinetics. A more robust interpretation is that structure and kinetics are tightly linked—efficiently packed structures are expected to have reduced kinetics and fragility. This has previously been demonstrated^35^, but a quantitative connection between structure and kinetics is still missing. A clue is offered by studies on Cu–Zr metallic glasses—packing efficiency (density) was found to be important in defining compositions with best GFA^45^. Subsequent analysis suggested that other properties such as expansivity are important^46^, but more recent work strengthens the connection between structure and kinetics by demonstrating a direct relationship between structural ordering and kinetic fragilities in metallic glasses^47^. This connection between atom sizes and kinetics supports the foresight of Cahn^48^, a well-respected metallic glass researcher and a pioneer in establishing the field of materials science, who predicted that, “simple geometry. . . atomic sizes. . . will prove to be the main criterion that in various subtle ways incorporates the others” . The present work is also able to explain the positive effect of small additions, 4–7 atom %, of large atoms on GFA (Supplementary Note 6).

Chemistry is expected to have a direct impact on GFA, since structures of chemically distinct atoms can have significantly different GFA. For example, BMGs form in the Zr–Cu system but not in Zr–Ni. Since Cu and Ni atoms are essentially the same size, this GFA difference is attributed to a chemical effect^49^. The full predictive power of the present model has therefore not yet been achieved, since it predicts the same GFA of structures regardless of their chemical make-up. Nevertheless, the present work gives a major new capability that is orders of magnitude more effective than current trial-and-error methods. It gives a necessary condition for good GFA, since every BMG we have compared against our model satisfies the present constraints. But it is not sufficient—a clear description of the chemical contribution is needed for a fully predictive model. The specific nature of this chemical effect remains an important, unsolved problem.

The present work resolves a seminal question in metallic glasses by establishing important details of the structures, derived from relative atom sizes and concentrations, needed to form BMGs. We identify new structural defects that significantly limit binary BMGs but have a less dramatic effect in more complex glasses. We show that BMGs require efficient local packing around all atom species and show how this is accomplished. Structural self-consistency gives constraints that are met by only a limited set of binary glass structures, but additional degrees of freedom in higher order systems dramatically expand the number of BMGs. The present model thus gives significant progress towards the long-sought goal of a predictive capability for the most stable metallic glasses. These conditions are necessary but not sufficient and some feature is missing—this is suggested to be a chemical contribution that forms the remaining seminal question in metallic glass stability. And while some of the connections between structure, free volume, fragility, viscosity and GFA are emerging, a more quantitative connection between these features is still desired.

## Methods

### Critical concentration of super-substitutional defects

Consider A and B atoms in a face-centred cubic (fcc) crystal^33^,^34^ that competes with the metallic glass, where B is the minority (solute) species. A solute atom can replace *N*>1 solvent atoms when it is sufficiently large. For example, a tetrahedron of solvents (*N*=4) leaves a symmetric space that can be filled by a larger B atom. In addition to this tetrahedral ‘super-substitutional' defect, three other sufficiently symmetric defects occur within the radius ratios relevant for metallic glasses. These are listed in Supplementary Table 2, along with the radius ratios that just fill these super-substitutional defects.

Foppl notation^50^ gives a simple description of each defect configuration. As used here, (*n*_1_,*n*_2_…)_*hkl*_ is the number of atoms, *n*_i_, in successive parallel *h,k,l* planes of an fcc structure and Σ*n*_*i*_=*N*. Critical defect concentrations are calculated as in ref. 34. The strain given by each defect vanishes and the defect concentration, *f*_B_, approaches 1 at the *R*_A/B_ values shown. We truncate the defect concentration at *f*_B_=0.50 for convenience, the actual value may be different. This analysis gives the atom fraction of B atoms, *f*_B_, which is transformed to *f*_crit_=1—*f*_B_ in Fig. 1. A spike in the *f*_B_ versus *R*_A/B_ curve near the minimum in *f*_crit_ between competing defects is due to partial occupancy of both defects^34^. We eliminate this artifact by assuming only the defect with the lowest strain energy is occupied.

The *N*=3 defect is asymmetric but may also be important. The solute that just fits in this defect has *R*_B/A_=(4/√3)−1 within the plane of the defect and *R*_B/A_=1 normal to the defect plane. This gives an average *R*_B/A_ of 2/√3, so that an excluded region is expected near *R*_A/B_=√3/2=0.866. The averaging used here is only an approximation, but the result is included in Fig. 1 and Supplementary Fig. 2c,d for completeness.

This approach is for super-substitutional defects that are largely symmetric. We can represent the degree of asymmetry of a super-substitutional defect as the difference between the minimum and maximum sphere radii that can fit in the super-substitutional defect normalized by the size of the host sphere. A divacancy has an asymmetry factor of 100%—this is too asymmetric to be meaningful and such defects are not included here. Super-substitutional defects for clusters of four vacancies (a tetrahedron) and six vacancies (an octahedron) are symmetric and have asymmetry factors of 0%. Super-substitutional defects with 8 and 10 vacancies are nearly symmetric, and the most asymmetric super-substitutional defect used in the present work has three vacancies and an asymmetry factor of 30%.

### Radius ratios for A- and B-centred clusters

The effective radius ratio in *i*-centred clusters, 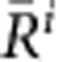, is


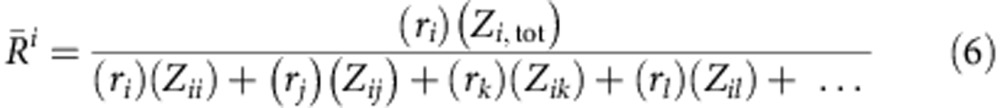


so that for binary A–B systems


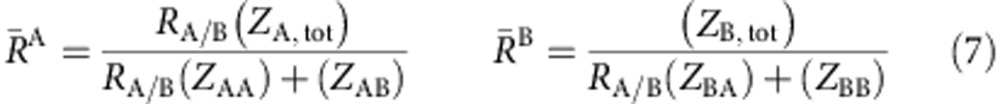


The packing efficiency of atoms in the first shell of an *i*-centred cluster is^30^


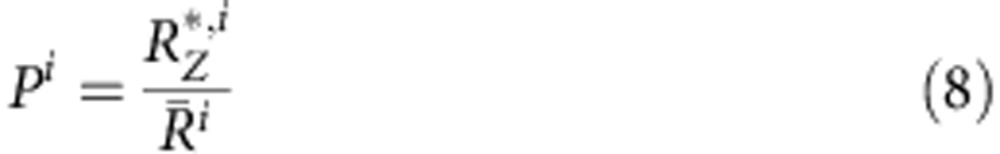


This extends an earlier approach for solute-lean clusters to solute-rich glasses and has been validated elsewhere^20^,^30^. Combining equation (7, 8) gives the nominal radius ratios of A- and B-centred clusters in equation (2).

### Simultaneous solution of structural self-consistency: binary glasses

We combine equations (2) and (3) to give


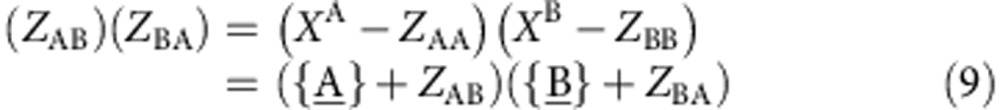


where 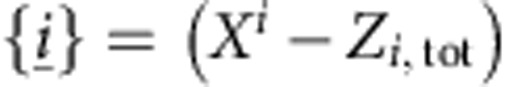 and 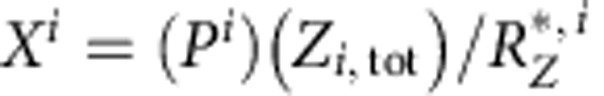. Expanding the product gives


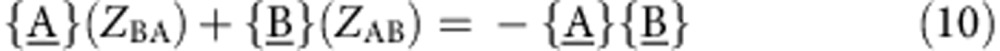


Two solutions exist for each intersection—one where 
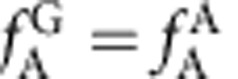
 (A-reference solution) and one where 
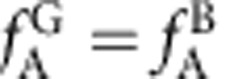
 (B-reference solution). For the A-reference solution, we substitute 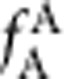 from equation (1) into equation (4) and rearrange terms to give


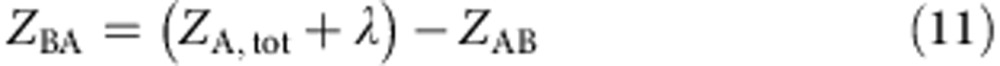


Rearranging equation (10) gives


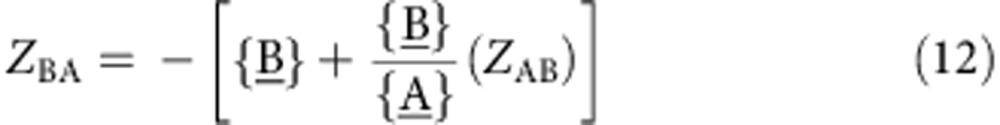


Setting equation (11) and (12) equal and solving for *Z*_AB_ gives


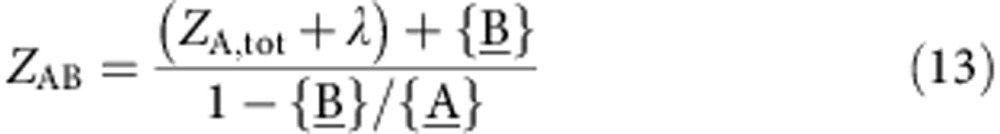


Using *λ*=2, *Z*_AB_ gives 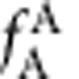 and 
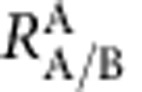
; we determine *Z*_BA_ from equation (4) and calculate 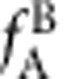 from equation (1).

The B-reference solution follows a similar approach to give the final solution,


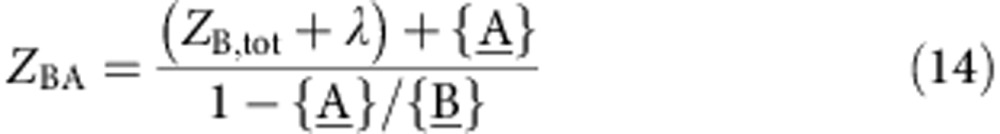


### Efficient packing in ternary metallic glasses

From equation (6), the effective radius ratios around A, B and C atoms are


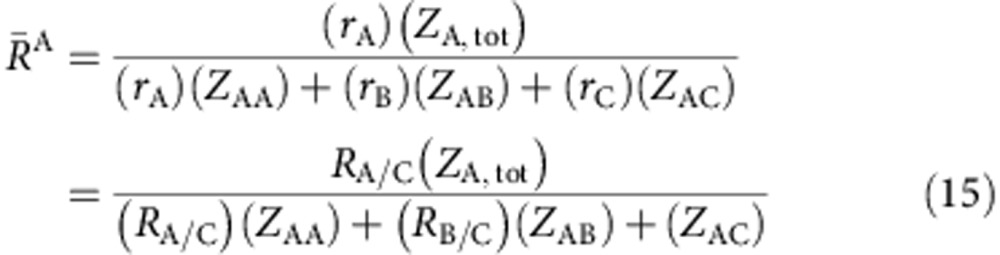



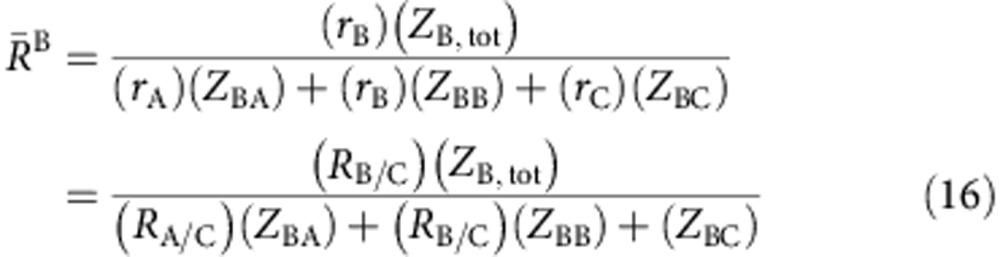



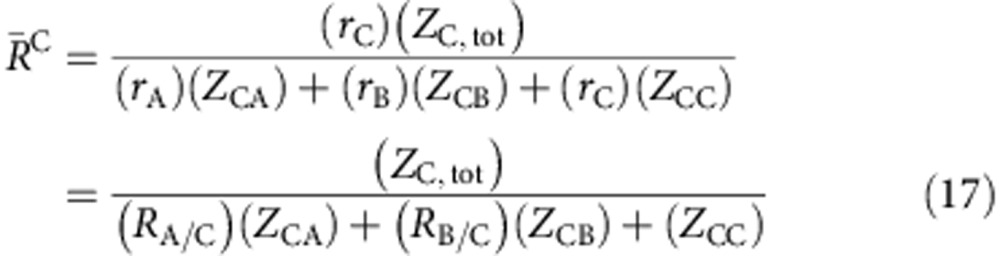


where *r*_A_<*r*_B_<*r*_C,_ and *R*_*i/j*_=*r*_*i*_/*r*_*j*_. Combining (
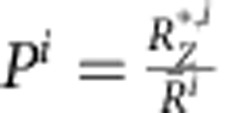
) and rearranging terms gives


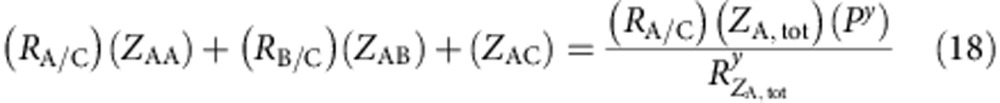



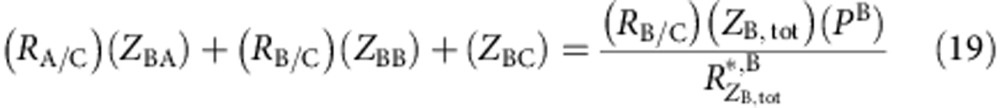



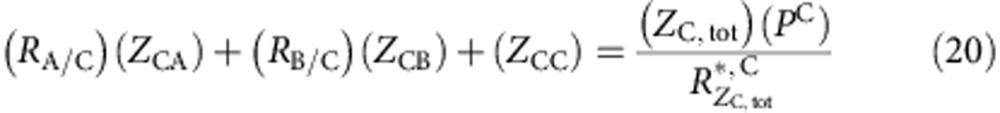


From equation (1)


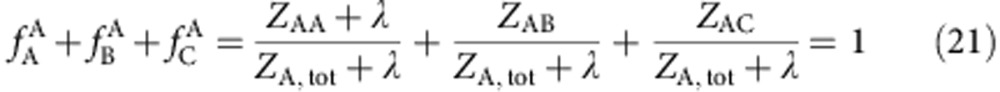



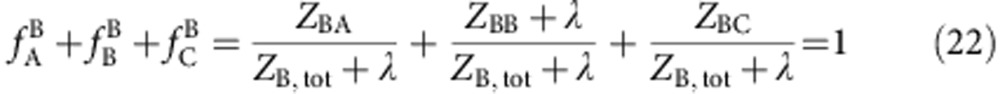



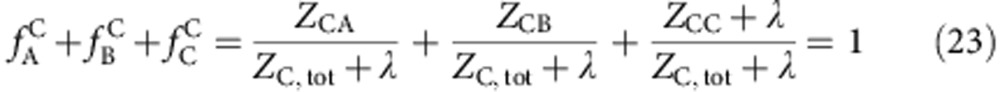


Efficiently packed clusters in the A−B−C ternary diagram have compositions along specific lines (Fig. 2). For A-centred clusters, the efficient packing line intercepts the A−C binary when *Z*_AB_=0 and *Z*_AA_=*Z*_A,tot_−*Z*_AC_. Inserting these into equation (18) and rearranging terms gives


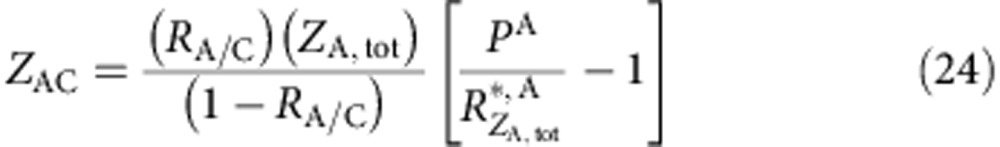


Combining with equation (21) and using 
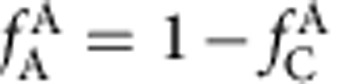
 gives


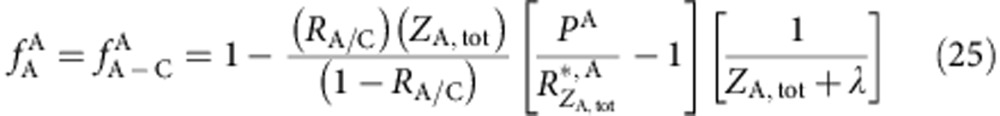


These values are given as 
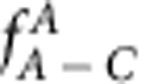
 in Supplementary Table 3, where the superscript is the atom at the cluster centre, the subscript is the binary line contacted by the intercept and the first letter in the subscript is the atom for which the concentration is given. Following a similar approach, efficiently packed A-centred clusters intercept the B–A binary boundary at


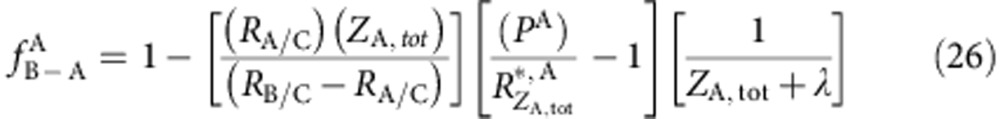


Using the same approach, intercepts for efficiently packed B-centred clusters are given by


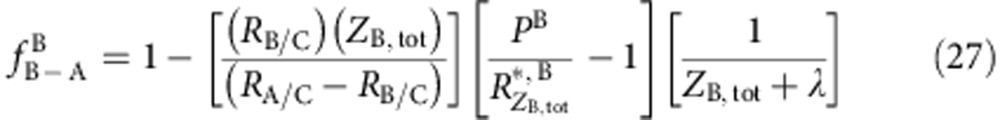



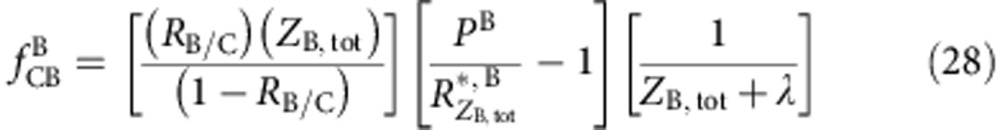


Finally, the intercepts for efficiently packed C-centred clusters are determined from


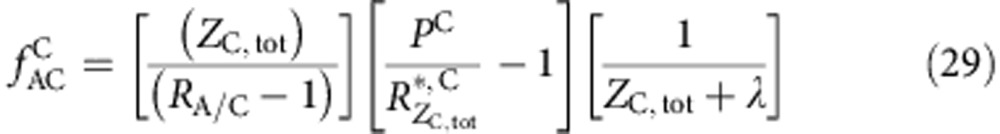



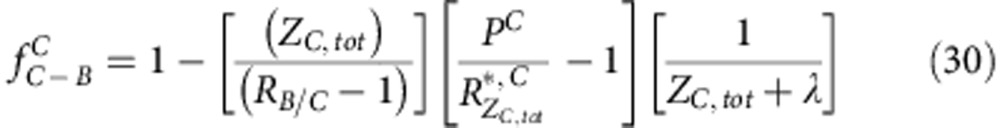


For a given <*Z*_A,tot_, *Z*_B,tot_, *Z*_C,tot_> structure, the efficient packing lines for A- and C-centred clusters both intersect the A−C binary boundary at the same composition using the *R*_A/C_ and *R*_B/C_ values in Supplementary Table 3. The same is true for efficient packing intercepts of A- and B-centred clusters on the A−B binary boundary and of B- and C-centred clusters on the B−C binary boundary. The compositions of A-centred clusters do not intersect the B−C binary boundary, B-centred clusters do not intersect the A−C boundary and C-centred clusters do not intersect the A−B boundary, since the minimum *i* atom fraction for structures of *i*-centred clusters is *f*_*i*_=1/(*Z*_*i*,tot_+*λ*). At these minimum compositions, the *j* atom fractions of *i*-centred clusters, 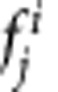, are


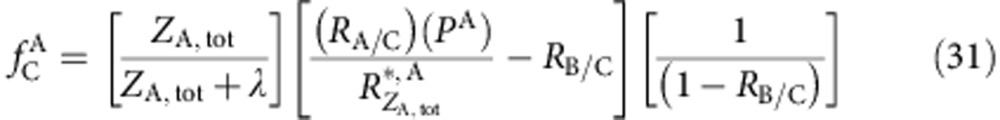



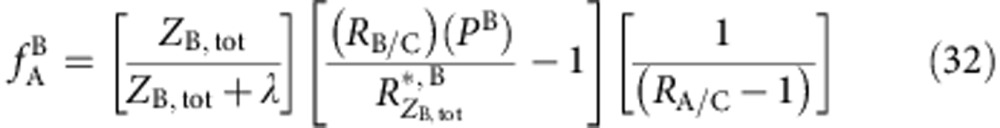



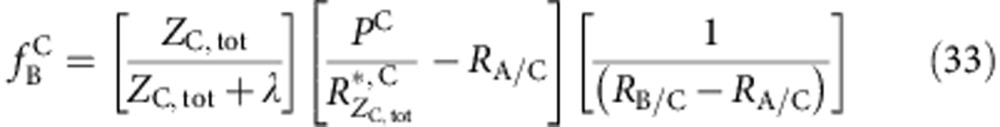


These compositions, together with *f*_*i*_=1/(*Z*_*i*, tot_+*λ*), lie on the efficiently packed lines given by equations (24, 25, 26, 27, 28, 29, 30). Thus, all three clusters for each given <*Z*_A,tot_, *Z*_B,tot_, *Z*_C,tot_> structure have the same efficiently packed lines. Using *P*^i^=1, *λ*=1 and the specific *R*_A/C_ and *R*_B/C_ values from Supplementary Table 3 for each <*Z*_A,tot_, *Z*_B,tot_, *Z*_C,tot_> structure gives the efficient packing intercepts in Fig. 2 and Supplementary Table 3. The shaded bands of efficient packing are obtained using *P*^*i*^=0.98 and *P*^*i*^=1.02.

### Structural self-consistency: ternary, quaternary and quinary glasses

Structural self-consistency provides three equalities in ternary systems


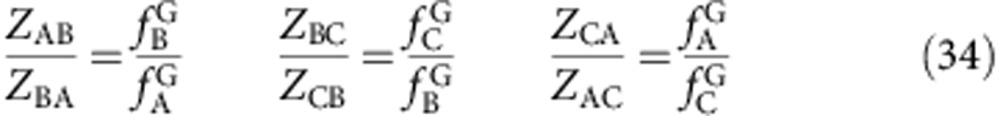


This gives the surprising result


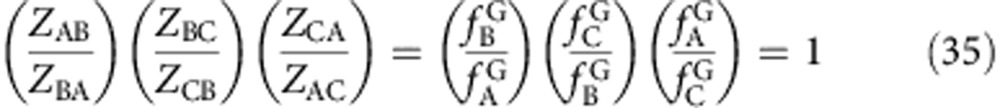


Using the *Z*_*ij*_ values in equation (34) for the glass compositions via equation (1) gives the same result. Thus, self-consistency is always satisfied in ternary glasses when the cluster composition is the same as the glass composition. Following a similar approach for quaternary alloys,


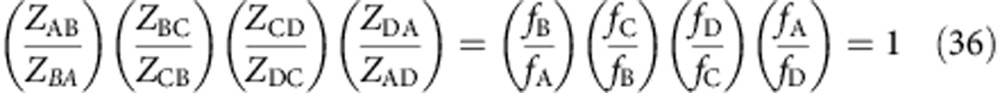


There are two solutions to self-consistency for quinary glasses


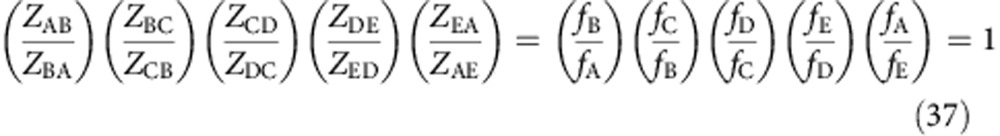



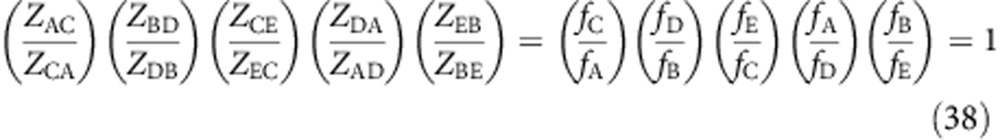


As for ternary glasses, self-consistency is always satisfied in quaternary and quinary glasses when the cluster composition is the same as the glass composition.

## Additional information

**How to cite this article**: Laws, K. J. *et al.* A predictive structural model for bulk metallic glasses. *Nat. Commun.* 6:8123 doi: 10.1038/ncomms9123 (2015).

## Supplementary Material

Supplementary Information Supplementary Figures 1-4, Supplementary Table 1-6, Supplementary Note 1-6 and Supplementary References

## Figures and Tables

**Table 1:** Simultaneous efficiently packed clusters (S-points) and their glasses.

***Z***_**A,tot**_	***Z***_**B,tot**_	**Ref. cluster**	***Z***_**AB**_	***Z***_**BA**_	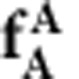	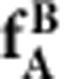	**Δ*****f***_**A**_	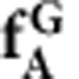	***R***_**A/B**_	***t***_**crit**_ **(mm)**	***R***_**A/B**_**(actual)**	Reported systems*
12	14	A	9.00	5.00	0.357	0.313	0.045	0.357	0.874	**1**	**0.873***0.899*	**Ti–Co (*****f***_**A**_**=0.30, 0.40)**Zr–Pd (*f*_A_*=0.30*)
10	14	A	9.08	2.92	0.243	0.182	0.061	0.243	0.783	**7–8**	**0.775***0.7970.797*	**Pd–Si (*****f***_**A**_**=0.18–0.20)**Zr–Cu (*f*_A_*=0.25–0.48)*Zr–Ni (*f*_A_*=0.25–0.40*)
11	14	A	8.60	4.40	0.339	0.275	0.063	0.339	0.856		*0.844*	Mg–Zn (*f*_A_*=0.35*)
12	16	B	5.35	12.65	0.618	0.703	0.085	0.703	0.804		*0.797*	Zr–Cu (*f*_A_*=0.72*)
12	15	B	7.02	9.98	0.499	0.587	0.088	0.587	0.843	**1–2**	**0.840**	**Nb–Ni (*****f***_**A**_**=0.60–0.62)**
12	14	B	10.34	5.66	0.261	0.354	0.092	0.354	0.888		*0.900*	Zr–Pd (*f*_A_*=0.30*)
12	15	A	5.65	8.35	0.596	0.491	0.105	0.596	0.813	**2**	**0.818**	**Ta–Ni (*****f***_**A**_**=0.59–0.62)**
11	16	B	5.72	12.28	0.560	0.682	0.122	0.682	0.798	**21.2–2**	**0.7970.797***0.7970.797*	**Hf–Cu (*****f***_**A**_**=0.65)Zr–Cu (*****f***_**A**_**=0.64, 0.645)**Zr–Cu (*f*_A_*=0.60–0.66*)Zr–Ni (*f*_A_*=0.63–0.67*)
11	15	B	7.41	9.59	0.430	0.564	0.134	0.564	0.837	**1–2**	**0.840**	**Nb–Ni (*****f***_**A**_**=0.60–0.62)**
11	15	A	5.49	7.51	0.578	0.442	0.136	0.578	0.792	**1–1.51**	**0.7970.797***0.797*	**Hf–Cu (*****f***_**A**_**=0.55, 0.60)Zr–Cu (*****f***_**A**_**=0.56, 0.60)**Zr–Cu (*f*_A_*=0.55*)
10	15	A	6.44	5.56	0.463	0.327	0.136	0.463	0.719		*0.715*	Zr–Be (*f*_A_*=0.30*—*0.50*)
11	14	B	10.68	5.32	0.178	0.332	0.154	0.332	0.881		*0.844*	Mg–Zn (*f*_A_*=0.35*)
10	15	B	9.52	7.48	0.206	0.440	0.233	0.440	0.791	**1.2–2**	**0.797***0.7970.797*	**Zr–Cu (*****f***_**A**_**=0.45, 0.46, 0.50)**Zr–Cu (*f*_A_*=0.25–0.48*)Zr–Ni (*f*_A_*=0.25–0.40*)
9	16	B	9.28	8.72	0.157	0.485	0.328	0.485	0.716		*0.715*	Zr–Be (*f*_A_*=0.30*—*0.50*)
9	15	B	10.83	6.17	0.015	0.363	0.348	0.363	0.747	**1**	**0.734***0.7340.703*	**Ca–Al (*****f***_**A**_**=0.336)**Ca–Al (*f*_A_*=0.35*)Ca–Zn (*f*_A_*=0.35*)

The <9,14>_A,B_, <9,15>_A_, <9,16>_A_, <10,14>_B_, <10,16>_A,B_, <11,16>_A_ and <12,16>_A_ structures are either non-physical or are suppressed by the *ɛ*_crit_ analysis and are not shown.

^*^Glasses in bold font are BMGs and all other glasses have Δ*T*_x_≥10 K. Data are taken from refs 32, 51.
