# Supplementary material for: A predictive structural model for bulk metallic glasses
Source: Nat Commun. 2015 Sep 15;6:8123. doi: 10.1038/ncomms9123 (PMC4648055; doi:10.1038/ncomms9123)
Supplement: Supplementary Information — Supplementary Figures 1-4, Supplementary Table 1-6, Supplementary Note 1-6 and Supplementary References [file ncomms9123-s1.pdf]

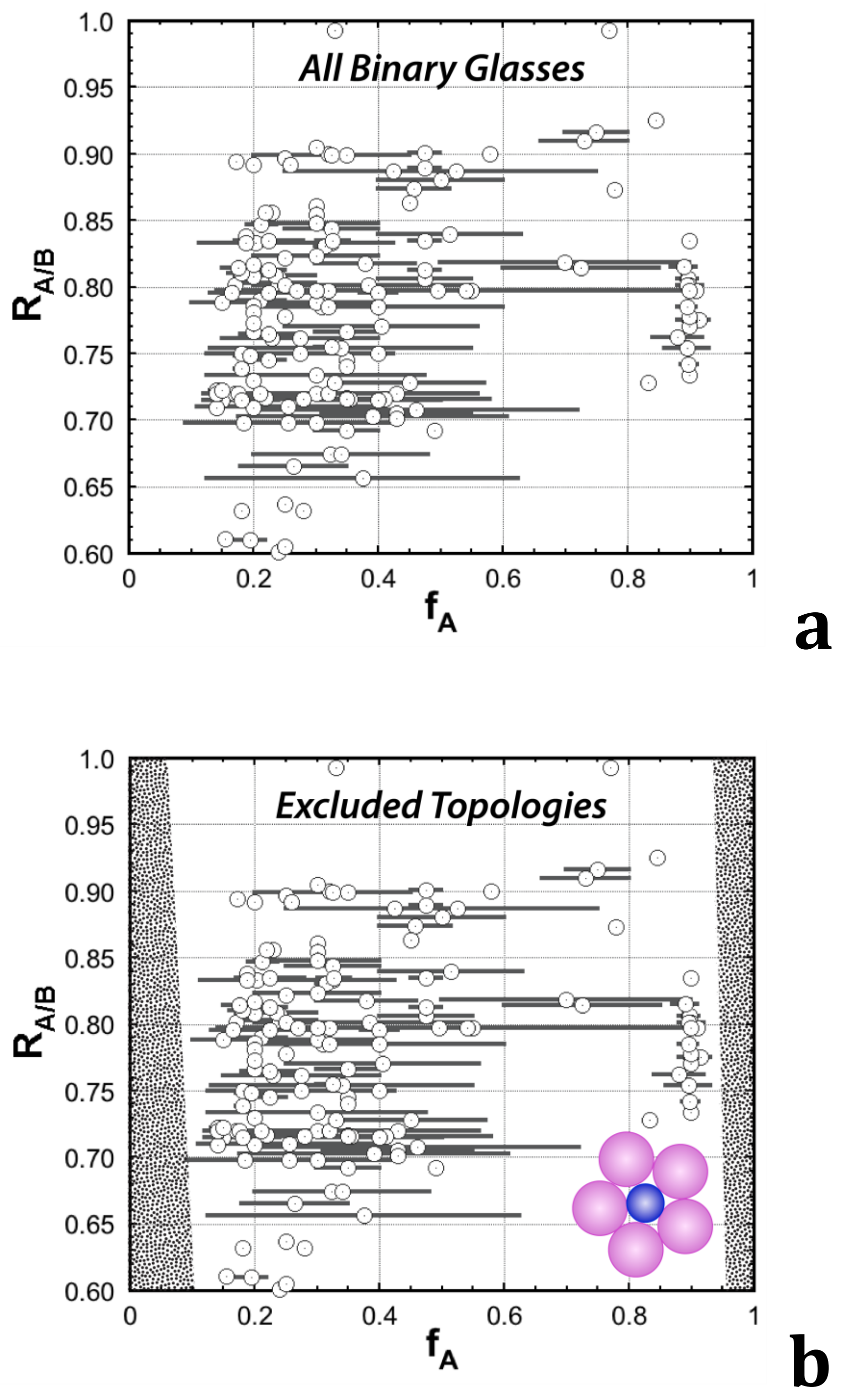

Supplementary Figure 1 (a) All binary metallic glasses. Each symbol represents a specific binary system of atoms, and the horizontal bar shows the compositional range over which that binary system forms a glass upon quenching from the liquid. (b) The relative atom sizes and concentrations excluded from forming metallic glasses are shown by the gray bands, and the inset illustrates solute-centered clusters with solvent atoms only in the 1st coordination shell. The data are taken from [2].

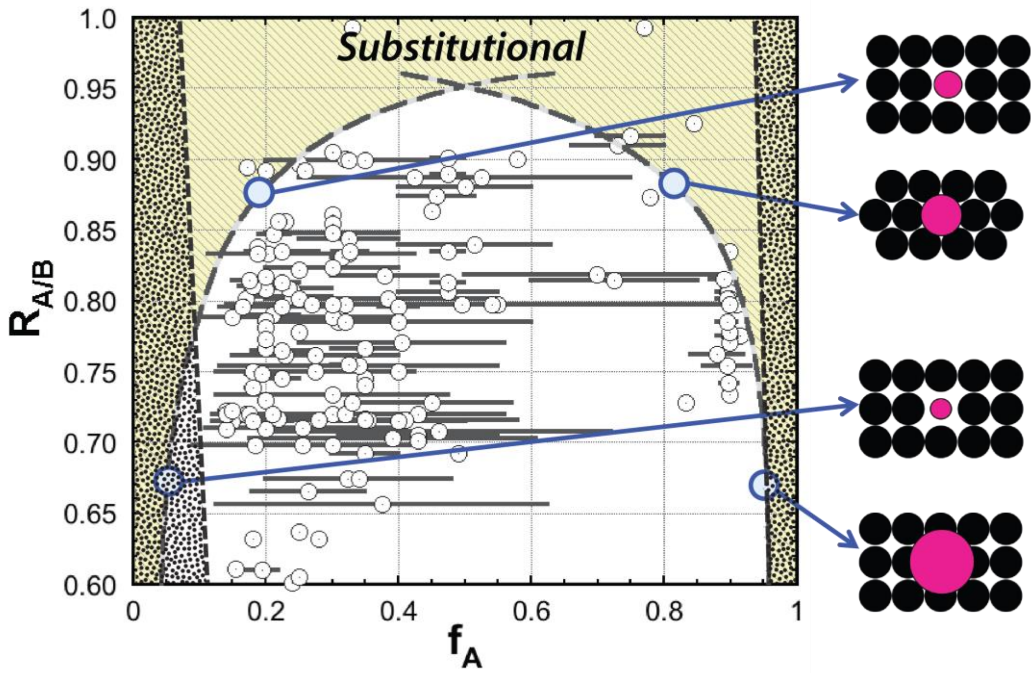
Substitutional
1.0
0.95
0.90
0.85
0.80
0.75
0.70
0.65
0.60
$R_{A/B}$
0
0.2
0.4
0.6
0.8
1
f_A

a

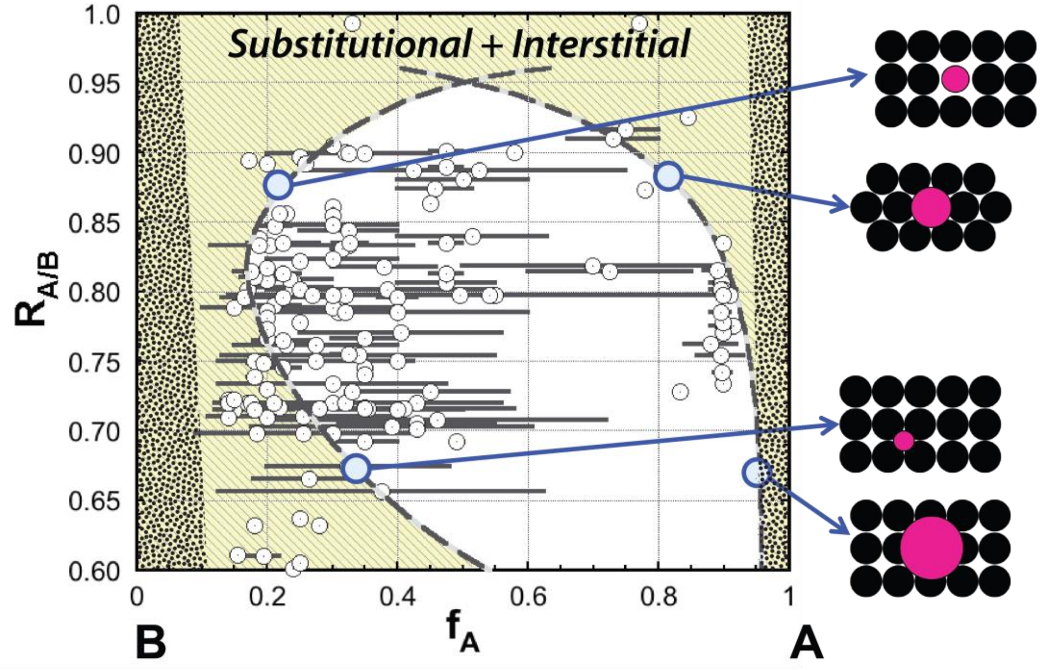
Substitutional + Interstitial
1.0
0.95
0.90
0.85
0.80
0.75
0.70
0.65
0.60
$R_{A/B}$
0
0.2
0.4
0.6
0.8
1
f_A
B
A

b

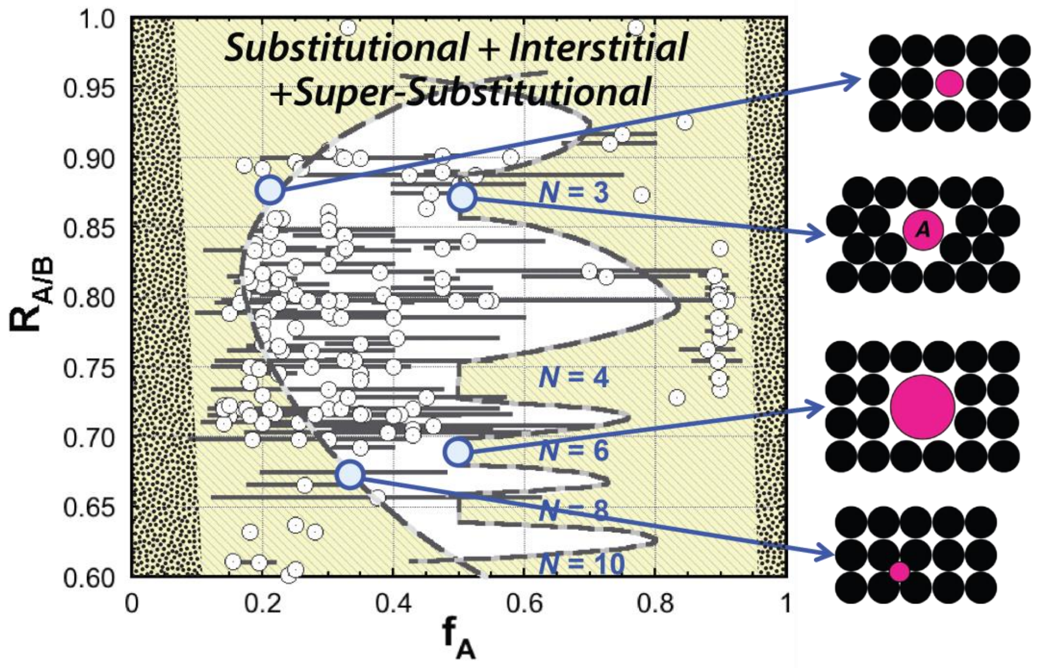

c

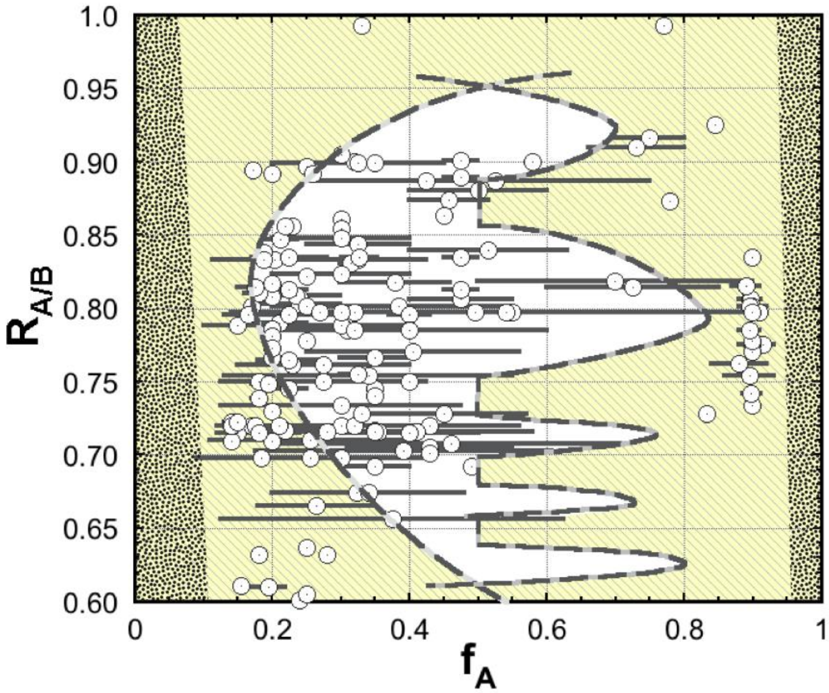

d

Supplementary Figure 2 Sequential builds showing discouraged regions in relative atom size – concentration space from substitutional, interstitial and super-substitutional point defects. Illustrations of the point defects responsible for each portion of the discouraged regions are also shown. The final result showing excluded (gray) and discouraged (yellow) regions is in panel d. See Supplementary Note 2 for discussion.

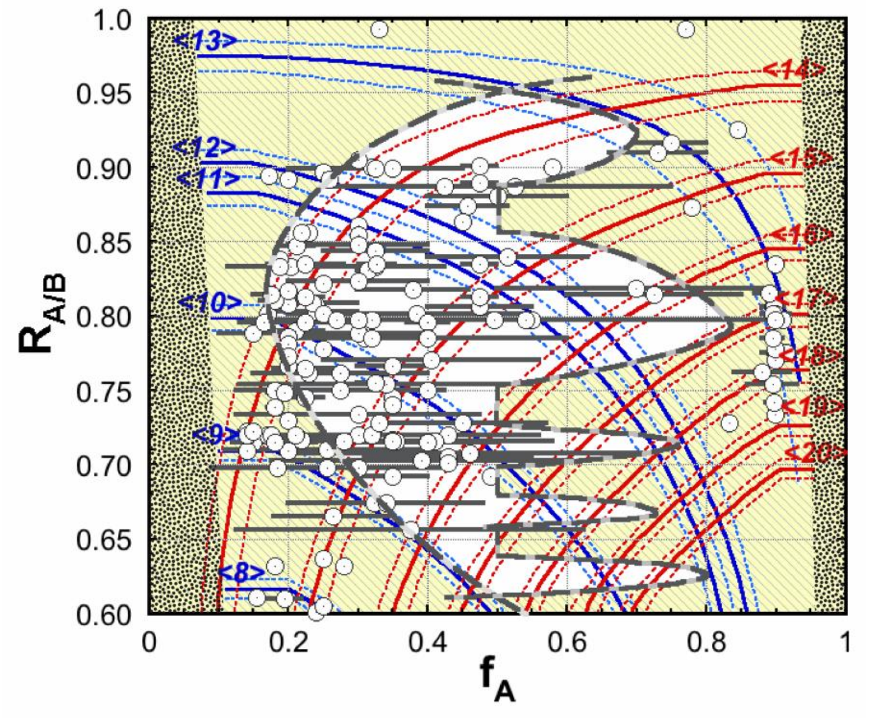

R A/B
f A
<13>
<12>
<11>
<10>
<9>
<8>
<14>
<15>
<16>
<17>
<18>
<19>
<20>

a

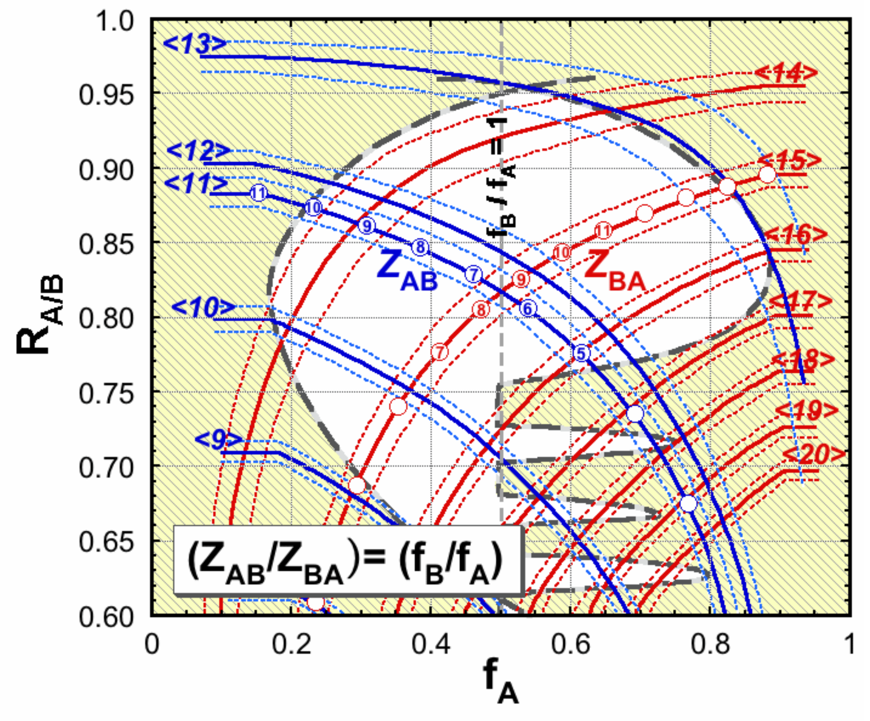

R A/B
f A
<13>
<12>
<11>
<10>
<9>
<14>
<15>
<16>
<17>
<18>
<19>
<20>
Z AB
Z BA
f B / f A = 1
(Z AB/Z BA)= (f B/f A)

b

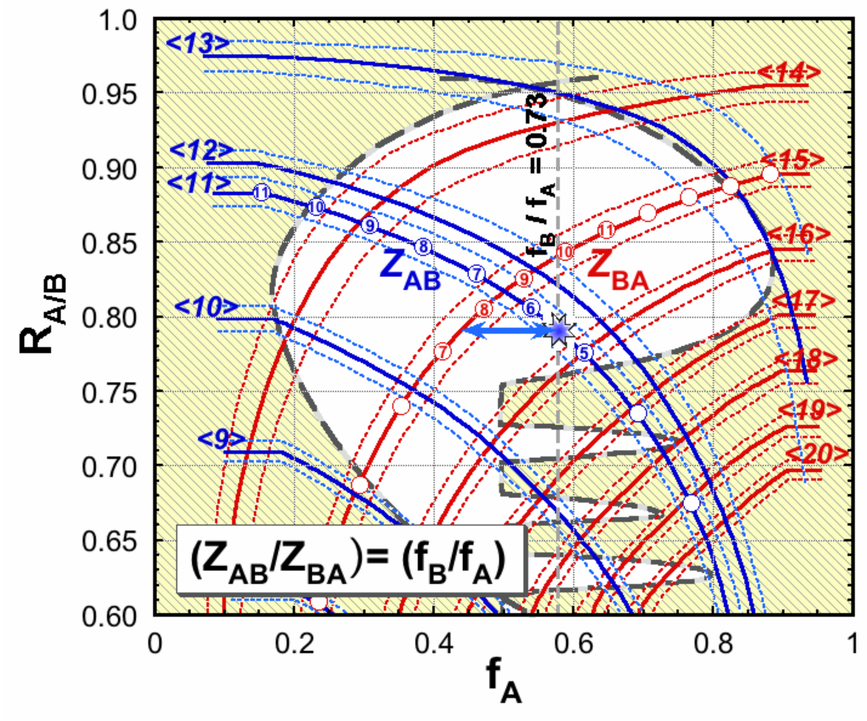

c

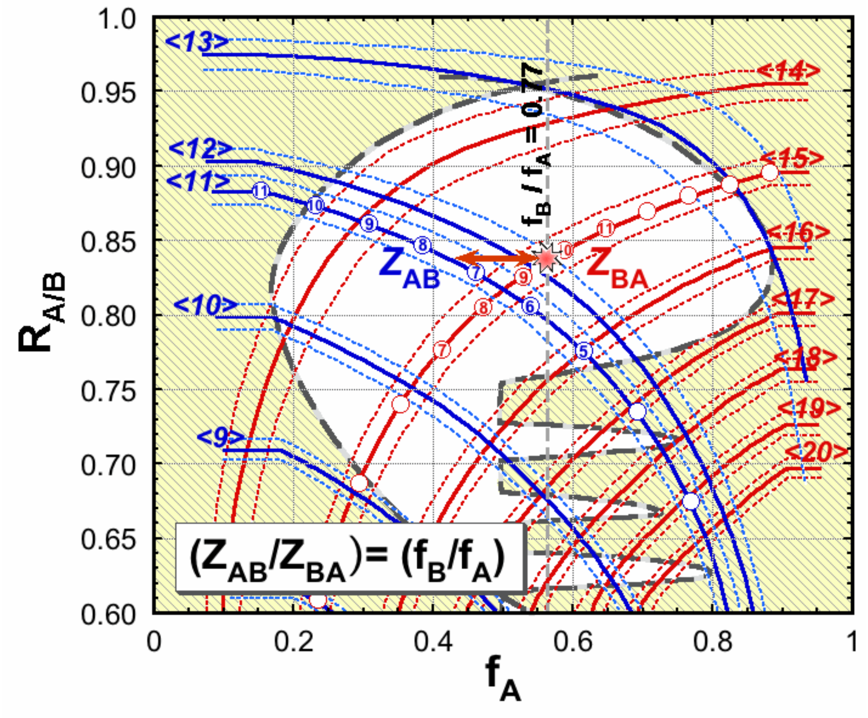

d

Supplementary Figure 3 Efficient cluster packing curves and a sequential build showing the simultaneous solution of the equations governing efficient atomic packing around both solute and solvent atoms in binary glasses. See Supplementary Note 3 for discussion.

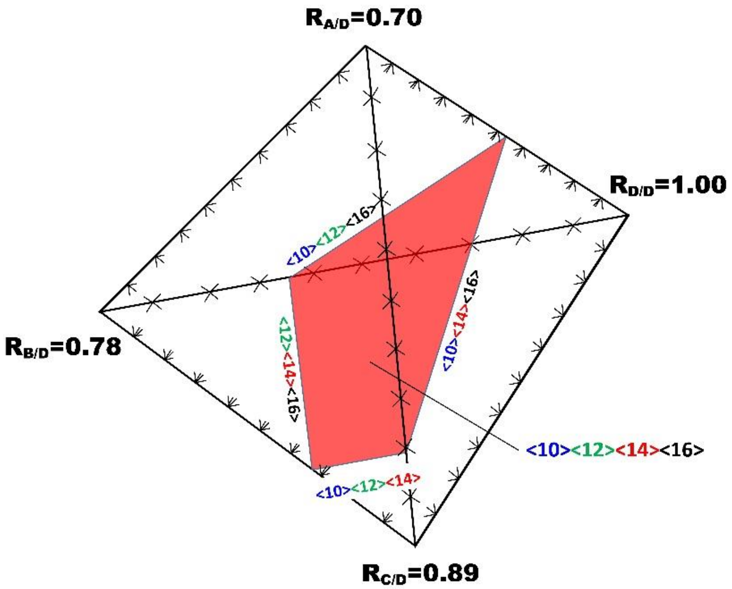

Supplementary Figure 4 Quaternary composition diagram. The ternary structural families <10,12,14>, <10,12,16>, <10,14,16> and <12,14,16> in Supplementary Table 5 are combined to show the efficiently packed <10,12,14,16> quaternary plane.

Supplementary Table 1 Atom radii used in the present work.

Atomic Number	Element Symbol	Atomic Radius [pm]	Atomic Number	Element Symbol	Atomic Radius [pm]	Atomic Number	Element Symbol	Atomic Radius [pm]
3	Li	152	34	Se	118	65	Tb	176
4	Be	116	37	Rb	244	66	Dy	175
5	B	88	38	Sr	212	67	Ho	177
6	C	77	39	Y	180	68	Er	175
7	N	72	40	Zr	158	69	Tm	175
8	O	64	41	Nb	150	70	Yb	190
11	Na	180	42	Mo	139	71	Lu	175
12	Mg	160	43	Tc	136	72	Hf	158
13	Al	140	44	Ru	134	73	Ta	154
14	Si	110	45	Rh	132	74	W	135
15	P	106	46	Pd	140	75	Re	137
16	S	103	47	Ag	144	76	Os	135
19	K	230	48	Cd	157	77	Ir	136
20	Ca	197	49	In	155	78	Pt	139
21	Sc	162	50	Sn	155	79	Au	143
22	Ti	142	51	Sb	155	80	Hg	152
23	V	134	52	Te	140	81	Tl	172
24	Cr	130	55	Cs	264	82	Pb	174
25	Mn	132	56	Ba	223	83	Bi	162
26	Fe	125	57	La	187	84	Po	168
27	Co	125	58	Ce	182	90	Th	178
28	Ni	126	59	Pr	183	91	Pa	165
29	Cu	126	60	Nd	182	92	U	158
30	Zn	139	61	Pm	185	93	Np	175
31	Ga	134	62	Sm	185	94	Pu	175
32	Ge	114	63	Eu	196			
33	As	115	64	Gd	176			

Supplementary Table 2 Super-substitutional Defects

N	Föppl Notation	$R_{B/A}$	$R_{A/B}$
3	$(3)_{111}$	$2/\sqrt{3}$	0.866
4	$(1,3)_{111}$	$\sqrt{11/2}-1$	0.743
6	$(3,3)_{111}$, $(1,4,1)_{100}$	$\sqrt{6}-1$	0.690
8	$(1,4,3)_{111}$	$\sqrt{58/9}-1$	0.650
10	$(3,4,3)_{111}$	$\sqrt{7}-1$	0.608

Supplementary Table 3 Structural ternary glasses $\langle Z_{A,tot}, Z_{C,tot}, Z_{C,tot}\rangle$ along with radius ratios $R_{A/C}$ and $R_{B/C}$ and intercepts of the efficiently packed cluster lines.

$Z_{A,tot}$	$Z_{B,tot}$	$Z_{C,tot}$	$R_{A/C}$	$R_{B/C}$	f^{A}_{A-C}	f^{B}_{A-B}	f^{C}_{B-C}	Reported glass systems in this composition range*
8	10	16	0.550	0.695	0.324		0.524	-
8	11	16	0.550	0.762	0.324		0.389	*Mo-[Si,P]-C, Zr-[Ni,Fe]-B*
8	12	16	0.550	0.776	0.324		0.324	
8	10	15	0.581	0.734	0.233		0.634	*Fe-B-N*
8	11	15	0.581	0.806	0.233		0.500	-
8	12	15	0.581	0.819	0.233		0.459	
8	10	14	0.616	0.779	0.110		0.810	***Pd-Si-B,*** *[Nb,Mo]-Si-B*
8	11	14	0.616	0.854	0.110		0.711	***[Fe,Ni,Co]-[Si,P]-C***
8	12	14	0.616	0.870	0.110		0.676	
9	10	16	0.625	0.695	0.388		0.524	*Fe-B-C*
9	11	16	0.625	0.762	0.388		0.389	*[Nb,Mo]-[P,Si]-B*
9	12	16	0.625	0.776	0.388		0.324	
9	13	16	0.625	0.835	0.388		0.119	***Ca-Mg-[Cu,Ni]***
9	14	16	0.625	0.891	0.388	0.778		***[Fe,Ni,Co]-Nb-B***
9	10	15	0.660	0.734	0.287		0.634	***Ca-Al-Cu, Sr-Mg-Zn***
9	11	15	0.660	0.806	0.287		0.500	***Ce-Al-[Cu,Ni,Co]***
9	12	15	0.660	0.819	0.287		0.459	***Ca-Mg-[Cu,Ni], Yb-Mg-Cu***
10	12	17	0.661	0.737	0.554		0.285	-
10	13	17	0.661	0.793	0.554		0.088	***Cu-Mg-[Ca,Yb]***
10	14	17	0.661	0.847	0.554	0.811		
10	15	17	0.661	0.900	0.554	0.632		-
10	12	16	0.695	0.776	0.478		0.324	*[Ce,La]-[Ni,Cu]-Al*
10	13	16	0.695	0.835	0.478		0.119	-
10	14	16	0.695	0.891	0.478	0.811	-	***Mg-[Cu,Ni]-[Y,Gd,La,Ce,Nd,Pr] Zr-Ti-Be***
9	10	14	0.700	0.779	0.141		0.810	*Zr-[Co,Cu,Ni]-Si*
9	11	14	0.700	0.854	0.141		0.711	*[Fe,Ni,Co]-[P,Si]-B*
9	12	14	0.700	0.870	0.141		0.676	
11	13	17	0.725	0.793	0.683		0.088	***Mg-[Ag,Pd,Zn]-[Ca, Yb]***
11	14	17	0.725	0.847	0.683	0.712		***Mg-Zn-Ca***
11	15	17	0.725	0.900	0.683	0.501		-
10	12	15	0.734	0.819	0.368		0.459	***Ca-Mg-[Al,Ag,Zn], Zr-Cu-Be, [La,Ce,Gd]-Al-[Cu,Co,Fe,Ni], Yb-Mg-Zn***
10	13	15	0.734	0.882	0.368		0.175	***[Pd,Pt]-[Cu,Fe,Ni]-[P], Au-Cu-Si***
12	14	17	0.738	0.847	0.717	0.678		***Mg-Zn-Ca***
12	15	17	0.738	0.900	0.717	0.458		***Ni-Nb-[Sn,Zr]***
11	13	16	0.763	0.835	0.614		0.119	-
11	14	16	0.763	0.891	0.614	0.712		*Zr-Cu-Al*
12	14	16	0.776	0.891	0.652	0.678		***[Zr,Hf]-[Cu,Co,Fe,Ni]-[Ag,Al,Pd,Ti], Au-Pb-Sb***
10	12	14	0.779	0.870	0.191		0.676	***Pd-[Cu,Fe,Ni]-[Si], Zr-[Cu,Fe,Ni]-Ti***
11	13	15	0.805	0.882	0.502		0.175	***[Zr,Hf]-[Cu,Co,Fe,Ni]-[Ag,Al,Ti]***

*Alloy systems shown in bold font form bulk metallic glasses

Supplementary Table 4 Thirty-five new BMGs that were first predicted by approaches in the present paper and then produced experimentally. Alloys were prepared by melting high-purity metals (≥99.8 wt.%) in graphite crucibles using an induction furnace in an argon-purged (99.997 wt.%) atmosphere. Rods were vacuum cast with diameters ranging from 1-4mm. The amorphous structure of each alloy was verified on the central section of rods using X-ray diffraction. Glass transition temperatures were determined by differential scanning calorimetry using a Netzch 204F1 calorimeter at a heating rate of 20 °C min^{-1}. A total of 44 compositions were predicted in these four systems, 35 of these were found to produce BMGs. To be published separately elsewhere.

**Mg-Ag-Ca**	**Mg-Ag-Yb**	**Mg-Pd-Ca**	**Mg-Pd-Yb**
$Mg_{75}Ag_{15}Ca_{10}$	$Mg_{76.7}Ag_{15.4}Yb_{7.7}$	$Mg_{72.5}Pd_{15}Ca_{12.5}$	$Mg_{77.5}Pd_{15}Yb_{7.5}$
$Mg_{75}Ag_{20}Ca_{5}$	$Mg_{75}Ag_{15}Yb_{10}$	$Mg_{72.5}Pd_{17.5}Ca_{10}$	$Mg_{75}Pd_{17.5}Yb_{7.5}$
$Mg_{70}Ag_{20}Ca_{10}$	$Mg_{75}Ag_{17.5}Yb_{7.5}$	$Mg_{72.5}Pd_{20}Ca_{7.5}$	$Mg_{75}Pd_{15}Yb_{10}$
$Mg_{67.5}Ag_{22.5}Ca_{10}$	$Mg_{72.5}Ag_{17.5}Yb_{10}$	$Mg_{72.5}Pd_{22.5}Ca_{5}$	$Mg_{73.75}Pd_{16.25}Yb_{10}$
	$Mg_{72.5}Ag_{20}Yb_{7.5}$	$Mg_{70}Pd_{17.5}Ca_{12.5}$	$Mg_{72.5}Pd_{20}Yb_{7.5}$
	$Mg_{70}Ag_{20}Yb_{10}$	$Mg_{70}Pd_{20}Ca_{10}$	$Mg_{72.5}Pd_{18.75}Yb_{8.75}$
	$Mg_{70}Ag_{22.5}Yb_{7.5}$	$Mg_{70}Pd_{22.5}Ca_{7.5}$	$Mg_{72.5}Pd_{17.5}Yb_{10}$
	$Mg_{67.5}Ag_{25}Yb_{7.5}$	$Mg_{67.5}Pd_{22.5}Ca_{10}$	$Mg_{72.5}Pd_{15}Yb_{12.5}$
		$Mg_{67.5}Pd_{25}Ca_{7.5}$	$Mg_{70}Pd_{20}Yb_{10}$
			$Mg_{70}Pd_{17.5}Yb_{12.5}$
			$Mg_{70}Pd_{15}Yb_{15}$
			$Mg_{67.5}Pd_{22.5}Yb_{10}$
			$Mg_{67.5}Pd_{20}Yb_{12.5}$
			$Mg_{67.5}Pd_{17.5}Yb_{15}$

Supplementary Table 5 Structural quaternary glasses $\langle Z_{A,tot}, Z_{B,tot}, Z_{C,tot}, Z_{D,tot}\rangle$ that form bulk metallic glasses in the given chemical systems and their associated $R_{A/D}$, $R_{B/D}$, $R_{C/D}$ values.

$Z_{A,tot}$	$Z_{B,tot}$	$Z_{C,tot}$	$Z_{D,tot}$	$R_{A/D}$	$R_{B/D}$	$R_{C/D}$	Reported glass systems in this composition range*
8	9	14	16	0.550	0.625	0.891	***[Fe,Ni,Co,Mn]-[Nb,Mo,W,Al]-B-C***
8	10	12	16	0.550	0.695	0.776	***[Fe,Ni,Co]-Zr-Si-B***
8	10	14	16	0.550	0.695	0.891	
8	11	14	16	0.550	0.762	0.891	***[Fe,Ni,Co,Mn]-[Nb,Mo,W,Al]-[Si,P]-C, [Fe,Ni,Co,Mn]-[Nb,Mo,W,Al]-Zr-B***
8	12	14	16	0.550	0.776	0.891	
9	10	13	17	0.600	0.661	0.793	***Ca-Mg-Zn-Cu***
9	10	14	17	0.600	0.661	0.847	***Ca-Mg-Zn-Cu***
9	10	15	17	0.600	0.661	0.900	
9	10	12	17	0.600	0.661	0.737	***Sr-Mg-Zn-Cu***
8	9	12	14	0.616	0.700	0.870	*[Fe,Ni]-P-B-C*
8	10	12	14	0.616	0.779	0.870	
9	10	12	16	0.625	0.695	0.776	
9	10	13	16	0.625	0.695	0.835	***Cu-Mg-Ca-[Ag,Zn]***
9	10	14	16	0.625	0.695	0.891	***[Fe,Ni,Co,Mn]-[Si,P]-B-C***
9	11	13	16	0.625	0.763	0.835	***Mg-Ag-Cu-Ca***
9	12	14	16	0.625	0.776	0.891	***[Fe,Ni,Co,Mn]-[Nb,Mo,W,Al]-Si-B***
9	10	12	15	0.660	0.734	0.819	***Ca-Mg-[Ag,Al,Zn]-Cu, Yb-Mg-Zn-Cu Sr-[Ca,Yb]-[Mg,Li]-Zn***
9	10	13	15	0.660	0.734	0.882	***Ni-Pd-P-B***
9	11	13	15	0.660	0.805	0.882	*Pd-[Cu,Ni]-Zr-P*
10	12	14	17	0.661	0.738	0.847	***Cu-Mg-Ca-Zn, Mg-[Ag,Pd]-Cu-Ca***
10	12	15	17	0.661	0.738	0.900	***Sr-[Ca,Yb]-[Mg,Li]-Zn Zr-Ti-[Cu,Ni]-Be***
10	12	14	16	0.695	0.776	0.891	***Ti-Zr-[Cu,Ni,Cr]-Be, Mg-[Cu,Ni]-[Ag,Zn]-[Gd,Y] Sc-Y-Al-Co***
9	10	12	14	0.700	0.779	0.870	***Zr-Pd-[Cu,Ni]-Si Zr-Ti-Cu-Be***

*Alloy systems shown in bold font form bulk metallic glasses

Supplementary Table 6 The known structural quinary BMGs <$Z_{A,tot}$, $Z_{B,tot}$, $Z_{C,tot}$, $Z_{D,tot}$, $Z_{E,tot}$> including those that form bulk metallic glasses in the given chemical and their associated $R_{A/E}$, $R_{B/E}$, $R_{C/E}$, $R_{D/E}$ values.

$Z_{A,tot}$	$Z_{B,tot}$	$Z_{C,tot}$	$Z_{D,tot}$	$Z_{E,tot}$	$R_{A/E}$	$R_{B/E}$	$R_{C/E}$	$R_{D/E}$	Reported BMG systems in this composition range
8	9	10	14	16	0.550	0.625	0.695	0.891	
8	9	11	14	16	0.550	0.625	0.762	0.891	*[Fe,Ni,Co]-[Nb,Mo,W,Al]-[Si,P]-B-C*
8	9	12	14	16	0.550	0.625	0.776	0.891	
8	9	10	12	14	0.616	0.700	0.695	0.870	
8	10	12	14	16	0.550	0.695	0.776	0.891	*[Fe,Ni,Co]-[Nb,Mo,W,Al]-Zr-[Si,P]-B*
9	10	12	14	16	0.625	0.695	0.776	0.891	
9	10	12	14	17	0.600	0.661	0.738	0.847	*Sr-[Ca,Yb]-[Mg,Li]-Zn-Cu*
9	10	12	15	17	0.600	0.661	0.738	0.900	

Supplementary Note 1: Detailed Description of Excluded Atom Sizes and Concentrations

To predict which alloys will form glasses, we must first understand why some alloys do not. Supplementary Figure 1 shows that all known binary glasses fill atom size – concentration space (atom fraction, f, and radius ratio between the smaller A and larger B atoms, $R_{A/B}$) unevenly. A minimum solute (minority species) atom fraction is needed to form a glass. Physically, this is the atom fraction at which each solvent atom (majority species) touches one and only one solute. The structure of this glass can be represented as consisting entirely of solute-centered atomic clusters with solvent atoms only in the 1st coordination shell. The solute atom fraction is $1/(Z+1)$, where Z is the number of solvent atoms in the 1st coordination shell. Z depends on $R_{A/B}$, and Z=12 when $R_{A/B} \cong 0.90$; Z=10 when $R_{A/B} \cong 0.80$ and Z=9 when $R_{A/B} \cong 0.71$ [1]. These values give the excluded zone when $f_A < 0.5$ in Supplementary Figure 1a. When the solute is larger than the solvent, Z=14 when $R_{B/A} \cong 1.05$; Z=17 when $R_{B/A} \cong 1.25$ and Z=20 when $R_{B/A} \cong 1.43$ [1]. The excluded zone for these systems is shown for $f_A > 0.5$ in of Supplementary Figure 1b by converting to $R_{A/B} = 1/R_{B/A}$ and giving the solute atom fraction of the larger atoms, f_B, as $(1-f_A)$. Supplementary Figure 1b shows that none of the reported binary metallic glasses fall in the excluded zones.

Supplementary Note 2: Detailed Description of Discouraged Atom Sizes and Concentration

Binary glasses are relatively uncommon when $R_{A/B} > 0.90$, when $R_{A/B} < 0.65$ or when $f_A > 0.6$ (Supplementary Figure 1). Metallic glasses must compete with crystals, and a critical strain analysis has been developed to show which relative atom sizes and concentrations are more likely to form crystals and may therefore be discouraged from forming glasses [3]. This analysis considers substitutional point defects in a competing face-

centered cubic (fcc) crystalline lattice. Each point defect generates a local strain field, and when a critical volume strain, ε_{crit}, is reached throughout the crystal, an amorphous phase may form. The smaller is the size difference between A and B atoms, the smaller is the local strain field and the atoms must be close together (have a high concentration) to reach ε_{crit}. As the size difference increases, the local strain also increases and the critical solute concentration, f_{crit}, needed to reach ε_{crit} also decreases. The results from this earlier analysis explains the sparsely populated regions in relative atom size – concentration speace when $R_{A/B} > 0.90$, but not for $R_{A/B} < 0.65$ or for $f_A > 0.6$ (Supplementary Figure 2a).

It is well-known that atoms will occupy interstitial sites in a crystalline lattice when they are sufficiently small. The largest interstices in an fcc lattice are octahedral interstices, which are perfectly filled (that is, strain-free) when the solute-to-solvent radius ratio is $\sqrt{2}-1 \cong 0.414$. Interstitial point defects can form at larger radius ratios, and will produce local strains that increase with increasing difference from the ratio 0.414. The strain energies of interstitial and substitutional defects are about the same when the radius ratio is ~0.81, so that interstitial defects will dominate in the competing fcc structure below this value [4]. The local strain decreases (and f_{crit} increases) as the solute size decreases. This new idea was used to expand discouraged regions in atom size – concentration space when $f_A < 0.5$ [4] (Supplementary Figure 2b). This gave a better coverage of sparsely populated regions, but the reason that glasses are uncommon with $f_A > 0.6$ remained a mystery.

In the present work, an entirely new point defect is introduced to explain this unsolved problem. These super-substitutional defects occur when the solute is sufficiently large relative to the solvent to displace more than one solvent atom. Five super-substitutional defects are expected when $0.6 \leq R_{A/B} \leq 1.0$, replacing N=3, 4, 6, 8 or 10 solvent atoms (see *Methods*). Each super-substitutional defect type gives a fairly narrow peak of discouraged atom sizes and concentrations, and these five newly discouraged regions account for

essentially all of the remaining sparsely populated binary regions in size –concentration space (Supplementary Figure 2c).

The final result is in Supplementary Figure 2d. In this chart, the gray regions are excluded structures where no metallic glasses are expected, the regions shaded yellow are discouraged, and the remaining areas of size – concentration space (white space) represents regions where good glasses can form. The agreement between observations and these predictions is good. No binary glasses occur in the excluded zones. The discouraged regions are sparsely populated, and the glasses in these regions are always marginal glasses (the maximum amorphous thickness, t_a, is less than 1 mm and the glass transition temperature, T_g, is essentially equal to the crystallization temperature, T_x). Additional insights into the structures of these discouraged glasses are given in the following section. The remaining atom sizes and concentrtations (white space) account for the majority of binary metallic glasses.

Supplementary Note 3: Detailed Description of Preferred Atom Sizes and Concentrations

Now that the excluded and discouraged structures are understood, we turn our attention to predicting those atom sizes and concentrations most likely to form metallic glasses. The efficient cluster packing (ECP) model is based on the idea of efficiently packed solute-centered clusters. Here we plot efficiently packed cluster curves in Supplementary Figure 3a for both solute- and solvent-centered clusters on the relative atom size - concentration plot from Supplementary Figure 2d. There is one efficiently packed cluster curve for each value of $Z_{i,tot}$ (the total number of atoms in the 1st shell surrounding an *i* atom). $Z_{i,tot}$ ranges from 8 – 20 for binary glasses. In each of these curves, $Z_{i,tot}$ is constant and the packing efficiency of atoms in the 1st shell of *i*-centered clusters, P^i, is 100%. Since

$r_A < r_B$, $Z_{A,tot}$ ranges from 8 – 13 (A-centered clusters, blue curves) and14 ≤ $Z_{B,tot}$ ≤ 20 (B-centered clusters, red curves). The horizontal portions of the efficient cluster packing curves are for 'solute-lean' clusters (those with solvent atoms only in the 1st coordination shell), and the remainder of the curves represents 'solute-rich' clusters (those with both solute and solvent atoms in the 1st shell). The dotted lines show the effect of changing the cluster packing efficiency by ±1%. This new idea shows the relative atom sizes and concentrations needed to give efficient packing in clusters of specified total coordination numbers, $Z_{i,tot}$. Additional details are given in Figure 1 of the main manuscript.

We notice that essentially all of the marginal glasses in the discouraged regions are efficiently packed around at least one of the clusters (Supplementary Figure 3a). This gives a simple, physical requirement to form discouraged glasses. We propose that the best glasses will have efficient packing around both A and B atoms simultaneously. It appears that this condition is met where two efficiently packed cluster curves cross. However, as discussed in the accompanying manuscript, two physical conditions must be satisfied simultaneously. First, the nominal radius ratio between A and B atoms in A-centered clusters, $R^A_{A/B}$, must be equal to the ratio between A and B atoms in B-centered clusters, $R^B_{A/B}$ (equation 3 in the manuscript). The second condition is called self-consistency, based on the fixed number of A-B bonds in the structure. This equation is $f^G_A Z_{AB} = f^G_B Z_{BA}$ (equation 4 in the manuscript), where f^G_i is the atom fraction of element *i* in the glass, and Z_{ij} is the number of *j* atoms in the 1st coordination shell of an *i* atom. These two equations are solved simultaneously in the accompanying manuscript (see *Methods*), here we give a visual illustration of how this simultaneous solution works.

Consider the intersection of $Z_{A,tot} = 11$ and $Z_{B,tot} = 15$ curves (Supplementary Figure 3b). This occurs exactly at $f_A = 0.5$, so that $f_B/f_A = 1$, and thus Z_{AB} must equal Z_{BA} to satisfy self-consistency. However, $Z_{AB} \cong 6.5$ and $Z_{BA} \cong 8.5$, so this condition is not met. We get closer to a solution by increasing f_A, and when $f_A \cong 0.58$, then $f_B/f_A = 0.72$ and $Z_{AB} \cong 5.5$. Since $R^A_{A/B} =$

$R^{B}_{A/B}$, we draw a horizontal line that passes through $Z_{AB} \cong 5.5$ and take Z_{BA} from the intersection of this line and the $Z_{B,tot} = 15$ efficient cluster packing curve. This gives $Z_{BA} \cong 7.5$, so that $Z_{AB}/Z_{BA} \cong 0.73$, which is nearly equal to $f_B/f_A = 0.72$. This is illustrated in Supplementary Figure 3c. This solution gives an S-point where the glass composition is the same as the composition of the A-centered clusters. The actual values for this <11,15>$_A$ S-point are shown in Table 1 of the accompanying manuscript. The B-reference S-point is obtained in a similar way at $f_A \cong 0.56$, $Z_{AB} \cong 7.5$ and $Z_{BA} \cong 9.5$ (Supplementary Figure 3d). Again, the actual values for the <11,15>$_B$ S-point are shown in Table 1. Thus, each intersection of efficient cluster-packing curves has two solutions – one where the glass composition is equal to the A-centered cluster composition and one where the glass composition is the same as that of B-centered clusters.

The agreement between the full set of predicted S-points and experimental observations is discussed in the accompanying manuscript.

Supplementary Note 4: Ternary BMGs

Supplementary Table 3 gives the predicted ternary families <$Z_{A,tot}$, $Z_{B,tot}$, $Z_{C,tot}$> in an A, B, C alloy system, where $r_A < r_B < r_C$. Reported metallic glass-forming systems that satisfy these specific radius ratios are also shown. Structures displayed in red are viable, but no glasses have been reported in these systems. Also given in Supplementary Table 3 are the radius ratios $R_{A/C}$ and $R_{B/C}$ and the intersection points on the binary axes that bound the ternary system at which efficient packing is achieved around each atom center. Using these intersection points, the efficient packing lines in Figure 2a-d can be constructed. For example, efficient packing around A, B and C atoms in the <10,12,15> system occur along the straight line between an A atom fraction on the binary A-C composition axis (f^{A}_{A-C}) of 0.368 and a C atom fraction on the binary B-C axis (f^{C}_{B-C}) of 0.459, as shown in Figure 2a.

Supplementary Note 5: Quaternary BMGs

Simultaneous efficient packing around all atom species occurs at distinct points for binary glasses, along lines for ternary glasses, and on planes for quaternary glasses. A specific efficiently packed plane for quaternary structural families can be generated using the intercepts given in Supplementary Table 3 for the ternary structures in the quaternary system (Supplementary Figure 4). Here, ternary diagrams like those in Figure 2 become the faces of a quaternary tetrahedron. Supplementary Figure 4 shows a tetrahdron for radius ratios $R_{A/D}$ = 0.70, $R_{B/D}$ = 0.78 and $R_{C/D}$ = 0.89 with the efficiently packed <10,12,14,16> quaternary plane. This plane is bounded by the ternary families <10,12,14>, <10,12,16>, <10,14,16> and <12,14,16>. Some planes can be described using only three families if they are in the corners of the tetrahedron. Knowing the values of f_A, f_B, f_C and f_D at the corners of the plane, the linear composition profile of the plane can be generated, giving the compositions of all efficiently packed alloys on this plane. Quaternary structures and reported glass systems are shown in Supplementary Table 4.

Supplementary Note 6: Large Minority Atoms and Improved GFA

Small additions can significantly improve GFA when the minority element is considerably larger than all other elements in the glass. The present model captures this in structures that consist completely of clusters centered by the largest atom. This occurs at an atom fraction of 1/(Z+1) as shown for $f_{A,min}$ earlier. Examples include Ca additions to Mg-Zn binary glasses with <11, 14, 17> structures and addition of Sn or Zr to Nb-Ni or Ta-Ni glasses with <12, 15, 17> structures. <*Z*> for the largest atom is 17 in these examples, giving a concentration of 5.6 atom%, which matches observed concentrations of 4-7% (see where the red shading starts near bottom of Figure 2a for $Mg_{46-75}Zn_{20-46}Ca_{4-7}$ glasses and

near the bottom of Figure 2c for $[Ni,Co]_{59-61}[Nb,Ta]_{34-38}[Sn,Zr]_{0-7}$). These clusters can also exist at lower large atom fractions, but with a lower abundance. Generally, major improvements in GFA are not reported until the constitution of the complete structural cluster unit is met. Given the relatively small addition of the largest atom, mathematically the effective radius ratio of the 1st coordination shell around the other clusters does not change greatly, but the relative radius ratios are shifted considerably relative to the much larger Ca atom. As a second effect, even a small addition of larger atoms is thought to slow crystallisation kinetics [45-47]. The smaller atoms need to 'go around' the larger atoms to rearrange into a crystalline lattice, thereby increasing diffusion distances of the smaller atoms. When the minimal addition of the large atom is met ($1/(Z+1)$), each of the smaller atoms is a 1st neighbour to a large atom, saturating this effect.

Supplementary References

1 Miracle, D. B., Sanders, W. S. & Senkov, O. N. The influence of efficient atomic packing on the constitution of metallic glasses. *Phil. Mag. A* **83**, 2409-2428 (2003).

2 Miracle, D. B., Louzguine-Luzgin, D., Louzguina-Luzgina, L. & Inoue, A. An assessment of binary metallic glasses: Correlations between structure, glass forming ability and stability. *Int. Mater. Rev.* **55**, 218-256 (2010).

3 Egami, T. & Waseda, Y. Atomic size effect on the formability of metallic glasses. *J. Non-Cryst. Sol.* **64**, 113-134 (1984).

4 Senkov, O. N. & Miracle, D. B. A topological model for metallic glass formation. *J. Non-Cryst. Sol.* **317**, 34-39 (2003).
